# Global Trends and Future Research Directions for Temporomandibular Disorders and Stem Cells

**DOI:** 10.3390/jfb14020103

**Published:** 2023-02-13

**Authors:** Zuleni Alexandre da Silva, Wallacy Watson Pereira Melo, Hadassa Helez Neves Ferreira, Rafael Rodrigues Lima, Renata Duarte Souza-Rodrigues

**Affiliations:** Laboratory of Functional and Structural Biology, Institute of Biological Sciences, Federal University of Pará, Belém 66075-110, Brazil

**Keywords:** temporomandibular joint, temporomandibular disorders, stem cells, tissue engineering

## Abstract

Temporomandibular disorder (TMD) is an umbrella term used to describe various conditions that affect temporomandibular joints, masticatory muscles, and associated structures. Although the most conservative and least invasive treatment is preferable, more invasive therapies should be employed to refractory patients. Tissue engineering has been presented as a promising therapy. Our study aimed to investigate trends and point out future research directions on TMD and stem cells. A comprehensive search was carried out in the Web of Science Core Collection (WoS-CC) in October 2022. The bibliometric parameters were analyzed through descriptive statistics and graphical mapping. Thus, 125 papers, published between 1992 and 2022 in 65 journals, were selected. The period with the highest number of publications and citations was between 2012 and 2022. China has produced the most publications on the subject. The most frequently used keywords were “cartilage”, “temporomandibular joint”, “mesenchymal stem cells”, and “osteoarthritis”. Moreover, the primary type of study was in vivo. It was noticed that using stem cells to improve temporomandibular joint repair and regeneration is a significant subject of investigation. Nonetheless, a greater understanding of the biological interaction and the benefits of using these cells in patients with TMD is required.

## 1. Introduction

The collective term temporomandibular disorder (TMD) encompasses a set of heterogeneous musculoskeletal and neuromuscular conditions that involves the masticatory muscles, the temporomandibular joint (TMJ), and/or the structures associated with them [1,2,3]. TMD is considered the main cause of non-dental pain in the orofacial region [1,2,4].

The prevalence of TMD varies between studies, depending on the studied population and the chosen evaluation method. However, a recent study [5] pointed out that TMD occurs in 31% of adults and the elderly. Another study showed that, although the prevalence of TMJ is higher in women, this difference between females and males is only marginally more significant [6].

The etiology of TMD is still controversial. However, studies show that its origin is multifactorial, including biopsychosocial factors [7,8]. Risk factors that exert influence for a few years before the manifestation of TMD signs and symptoms (predisponents) must be taken into consideration, as well as those that act for the condition to develop (initiators) and those that cause the continuation of TMD (perpetuators), making it difficult for the outcome of the treatment [9,10]. TMD signs and symptoms are varied and can include TMJ pain, masticatory muscle pain and/or fatigue, limited mouth opening, headache, joint noises (clicking, popping, or crepitus), TMJ locking, otalgia, tinnitus, ear fullness, and vertigo [9,10].

Due to the multifactorial etiology of TMD, several treatment strategies have been adopted and the choice of these varies according to the degree of severity of the disorder [11]. Preferably, more conservative methods have been chosen as the first line of treatment [12]. Among these, we can mention non-invasive ones such as patient education, pharmacotherapy, physiotherapy, interocclusal splints, prosthetic rehabilitation, and/or minimally invasive such as arthrocentesis, hyaluronic acid injections, intra-articular injections of corticosteroids, platelet-rich plasma (PRP), oxygen–ozone therapy, and arthroscopy [11,12,13,14]. In some cases considered as severe, when patients do not respond to the conservative treatments, more invasive and intra-articular surgical interventions are required [13,15].

From this perspective, tissue engineering with stem cells emerges as an interesting and promising regenerative therapy strategy. Stem cells can be derived from two main sources, which are embryonic stem cells (ESCs) and adult stem cells, including mesenchymal stem cells (MSCs), the most widely used in TMJ [16]. The use of these cells occurs at the process of repairing damaged tissues or those with impaired function, in order to replace and repair their normal physiology, in addition to suppressing and modulating the inflammatory process [11,15,17]. This would provide a decrease in painful symptoms, prevent progressive degeneration of cartilage and subchondral bone, therefore favoring the reestablishment of TMJ function [18]. 

Bibliometric studies are carried out to identify the existing knowledge on a given topic quantitatively and qualitatively, to clarify research trends, as well as highlight themes that are already obsolete and without relevance for the academic and scientific environment [19]. The quantitative data of these studies presents the intensity of research on the subject, such as the authors, journals, and countries that have published the most about it. Also, the number of citations and the impact they cause related to the quantity and quality of the productions and the construction of the knowledge are to be mapped [20]. In addition, bibliometric analysis can provide a current overview of a given subject and point out suggestions for new topics to be addressed and researched to fill the gaps in existing studies [19,21].

Bibliometric TMD mapping studies are already found in the scientific literature [12,22,23,24]. It was observed that they worked with the theme in general, sometimes selecting the most cited articles in each period. Bibliometric studies on stem cells were also found; however, these studies were focused on the mechanisms of action, characteristics, differentiation, and cell signaling [25,26].

To the best of our knowledge, no previously published bibliometric study has investigated the specific relationship of TMDs with the treatments currently used, nor with the use of stem cells for that purpose. Thus, the present study aimed to investigate research trends and indicate future directions for studies on TMD and stem cells.

## 2. Materials and Methods

A comprehensive search was performed on the main collection of the Web of Science database on 23 October 2022. Scopus and Google Scholar were used to compare the number of paper citations. Two independent researchers conducted the selection of articles, and cases of disagreement were resolved via consensus. Articles dealing only with TMD and stem cells were included, regardless of the TMD classification or the involved cell type. There was no restriction regarding the year of publication of the articles or the language. Nevertheless, as exclusion criteria, we considered letters to the editor, conference articles, and studies without abstracts. 

The search strategy used was elaborated with mesh terms related to the stem cells and temporomandibular joint (Supplementary file S1). Titles, year of publication, author’s name, number of citations, the density of citations, country, affiliations, abstract, journal’s name, study design, and author’s keywords were extracted from papers and imported into the program Microsoft Excel® and VOS viewer (CWTS, Leiden University, Leiden, The Netherlands; https://www.vosviewer.com/ (accessed on 23 October 2022)). With the VOS viewer, three images were generated: the first one was about the network of authors who produced the most on the subject, in which each node represented an author. The size and color of the nodes indicated the frequency of occurrence and to which group each author belonged. The links between each node showed the collaboration network between the authors. Thus, the thickness and proximity between them indicated more frequent collaborations. The second image is related to the articles’ country of origin. Each rectangle showed a country, their size pointed to the publications’ frequency, each color represented the period of greatest publication and the lines the collaboration networks formed between them. Again, the thickness of these and the proximity between the rectangles indicated more collaboration between countries. The third image is about the most cited keywords, in which the more intense the color and the larger the font size of the letters, the greater the occurrence density is. In contrast, the letters’ weaker colors and smaller font sizes indicate a lower occurrence.

After selection, the articles were read in full to search for information such as the type of stem cell used, tissue of origin of these cells, conditions caused in the TMJ, and proposed treatment method and biomarkers used. The main findings of in vitro studies were categorized according to cell origin, whether human or animal, the purpose of treatment, and the differentiation of stem cells. The biomarkers were organized according to their overexpression or inhibition, highlighting the possible influence of this result on TMD.

## 3. Results

**Figure 1:**
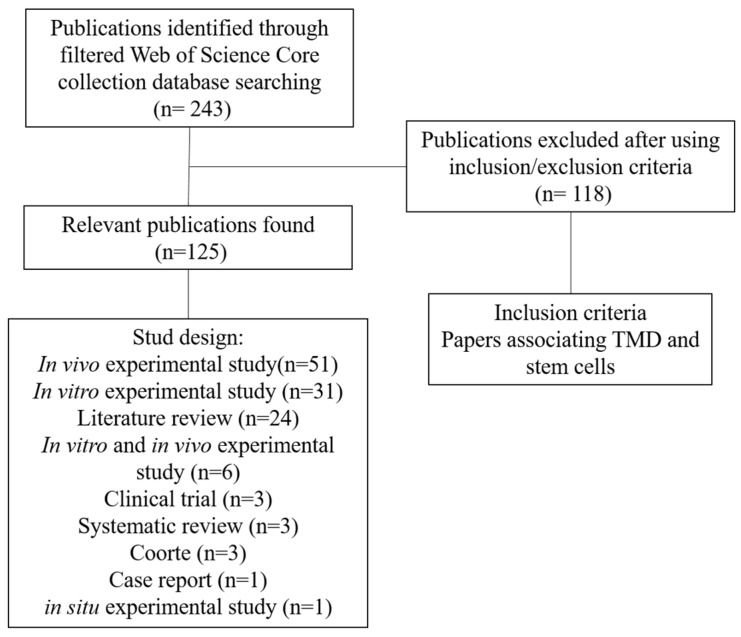
Flowchart of the article selection process on TMD and stem cells.

The search carried out in WoS-CC resulted in 243 articles, of which 125 were selected according to the eligibility criteria (Figure 1). The articles were organized according to the year of publication, from the oldest to the most recent (Supplementary file S2). The 125 articles selected in our study received a total of 1321 citations in WoS-CC, 1366 in Scopus, and 2103 in Google Scholar. It is worth mentioning that among the one hundred twenty-five articles, seven were not found in Scopus [27,28,29,30,31,32,33], and one was not found in Google Scholar [34].

The selected articles were published between 1992 and 2022. The oldest article, entitled “The relationship of undifferentiated mesenchymal cells to TMJ articular tissue thickness” by Bibb et al., 1992, [35] was published in the “Journal of Dental Research” and received 22 citations in WoS-CC (1.66%), 23 citations in Scopus (1.68%) and 43 citations in Google Scholar (2.04%). It aimed at evaluating the relationship between joint tissue thickness and the existence of undifferentiated cells in the TMJs of young adults. This study showed the beginning of investigations on the presence of stem cells in the joint region, which was an essential step for experiments currently being carried out that aim to characterize, differentiate, and treat TMJ disorders with undifferentiated or already-differentiated stem cells.

The most recent article, published in October 2022, was entitled “Stem Cells in Temporomandibular Joint Engineering: State of Art and Future Perspectives” and was published in the “Journal of Craniofacial Surgery” and received one citation in WoS-CC (0.075%), one in Scopus (0.073%), and three citations in Google Scholar (0.142%) [11]. It is a narrative review that brings several regenerative approaches to TMJs, including stem cell therapy. The authors report the existence and ability to extract mesenchymal stem cells from various tissues of the human body, including evidence of the ability of stem cells from fibrocartilage to form bone tissue in animal studies.

**Figure 2:**
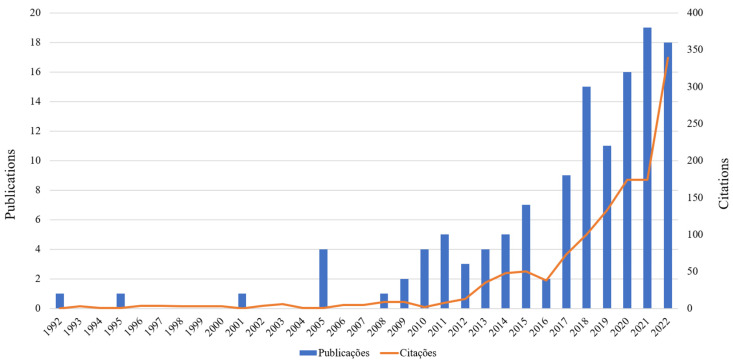
Distribution of articles published annually on TMD and stem cells, as well as their citations between 1992 and 2022.

The period in which the highest number of citations occurred was between 2012 and 2022 (n = 1.051; 79.56%). This was also the period when the highest number of articles were published (n = 109; 87.20%) (Figure 2). The most cited paper (67 citations in WoS-CC, 5,07%) was by Wu et al. in 2014, entitled “The pilot study of fibrin with temporomandibular joint derived synovial stem cells in repairing TMJ disc perforation” [36], and published in the journal “BioMed research international” which deals with a pilot study for the use of scaffold fibrin/chitosan hybrids and mesenchymal stem cells, to evaluate the repair of the TMJ joint disc. Twelve articles received only one citation [11,18,28,37,38,39,40,41,42,43,44,45]. Ten articles were not cited in Wos-CC [27,29,46,47,48,49,50,51,52].

A total of 65 journals were published on TMD and stem cells between the years 1992 to 2022. The ones that obtained the highest number of articles were the “Journal of Dental Research” (n = 11; 8.8%), “Biomed Research International” (n = 5; 4%), and the “International Journal of Molecular Science (n = 5; 4%). Among the studies published by the Journal of Dental Research, five articles were in vivo studies that aimed to characterize and stimulate stem cell differentiation [31,50,53,54,55]. Two were in vitro studies [56,57] and one in vivo/in vitro [58]; they also studied the cell differentiation and gene expression outcomes. The other three articles were review papers and aimed to elucidate the search for new therapeutic strategies for treating TMD and repair/regeneration of TMJ tissues [17,46,59].

**Figure 3:**
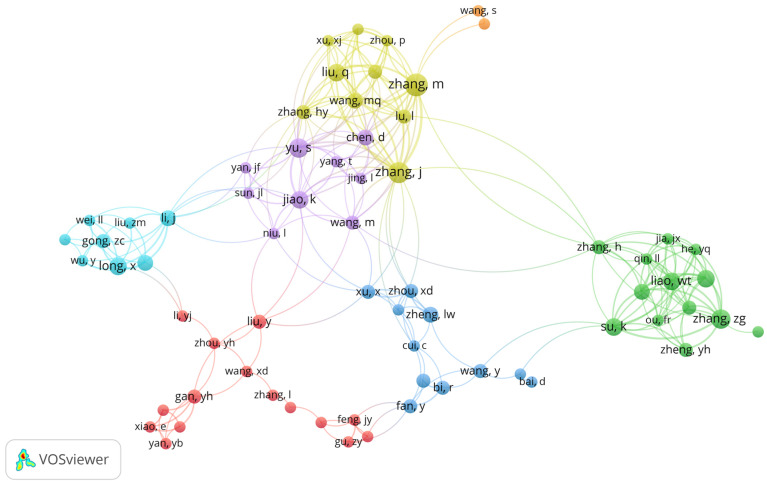
Network visualization of the authors who published the most on TMD and stem cells.

The authors with the most significant number of articles on the subject can be seen in Figure 3, which was elaborated considering a minimum of two publications per author. The authors with the highest number of published articles are Zhang, M with eight (6.4%) and Zhang, J with seven (5.6%). It was observed that these same authors shared co-authorship in six articles, demonstrating great interaction between them. In addition, it was identified that such authors corresponded with others from different research groups, such as YU, S (n = six; 4.8%) and JIAO, K (n = five; 4%).

Regarding the countries of origin of the articles, 34 were identified in total. The country with the highest number of published articles was China, with 61. The first publication took place in 2005 and the country has led in the number of publications since 2010. In turn, the USA ranked second, with 32 articles published from 1992 until 2022. Interestingly, the USA led in the number of publications only in the years 1992 (n = 1), 2001(n = 1), and 2009 (n = 2); years in which China did not publish any articles about TMD and stem cells.

**Figure 4:**
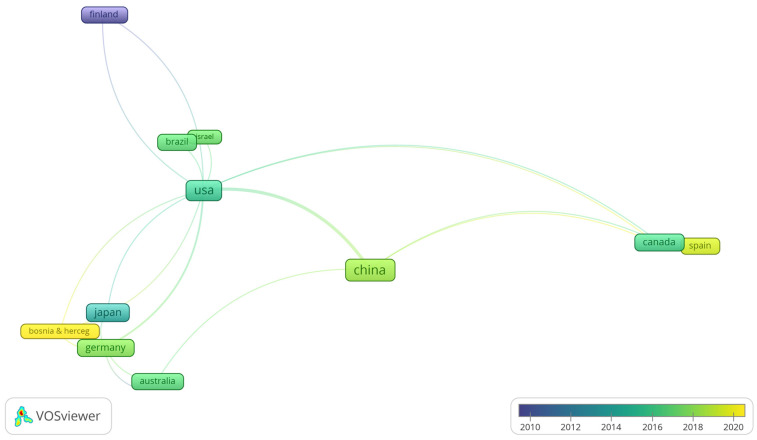
Countries with the highest number of articles published between 2010 and 2020.

Specifically, when analyzing Figure 4, we can see that from 2017 to 2019, China had a peak in publications, which led to a number of articles published. The USA also increased the number of articles published between 2015 and 2017 but remained in second place. Even so, Figure 4 shows that the US has the most connections with other countries, the ten most important being: China (n = sixty-one), Germany (N = five), Canada (n = three), Brazil (n = three), Finland (n = one), Israel (n = one), Saudi Arabia (n = one), and Japan and Bosnia (n = one).

**Figure 5:**
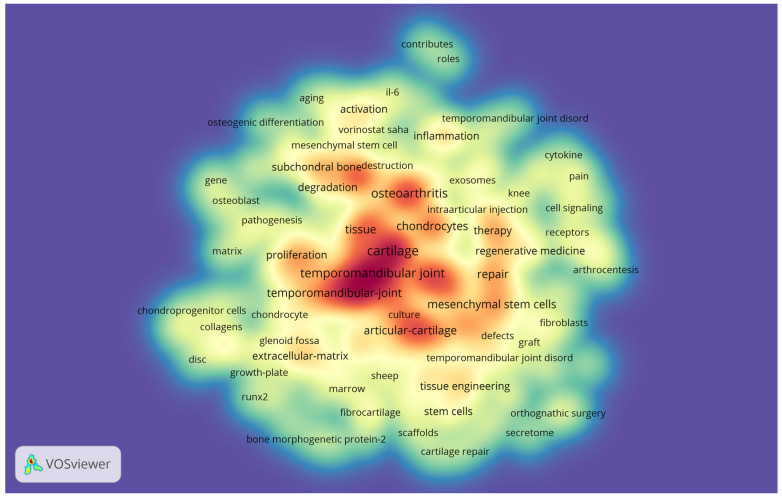
Keyword density map. Words most frequently found in the 125 selected articles. The colors indicate the citation density of the authors, ranging from blue (lowest density) to red (highest density).

184 keywords were found on the 125 selected articles (Figure 5). In that image, the warmest colors represent the highest occurrence of keywords in the articles. Thus, it was identified that the most used were “cartilage” (n = 41; 4.42%), “temporomandibular joint” (n = 36; 3.88%), “mesenchymal stem cells” (n = 33; 3.56 %), and “osteoarthritis” (n = 26; 2.80%). These words may be related to the most searched points of interest in the analyzed theme.

In vivo studies (n = 51; 40.8%; 574 citations in WoS-CC), followed by an in vitro study (n = 31; 24.8%, 274 citations in WoS-CC) and the literature review (n = 24; 19.2%, 234 citations in WoS-CC), were the three most frequent types of studies. Also, some papers performed both types of study (in vivo and in vitro) together (n = 6; 4.8%, 56 citations in WoS-CC). Clinical trial (n = three, twenty-three citations in WoS-CC), systematic review (n = three, twenty-five citations in WoS-CC), cohort (n = three, seventeen citations in WoS-CC), case report (n = two, four citations in WoS-CC) and in situ (n = two, sixteen citations in WoS-CC) were the least frequent types of studies, with 2.4%, 2.4%, 2.4%, 1.6%, and 1.6%, respectively.

Analyzing only the in vitro studies (n = 37; 30%), we observed that the mesenchymal stem cells collected for culture and cell differentiation were obtained both from humans and animals (Table 1). The sources of stem cells of animal origin were bone marrow stroma (n = 14; 37.8%), fibrocartilage (n = 1; 2.7%), synovial fluid (n = 1; 2.7%), and myelomonocytes (n = 1; 2.7%). As for human sources, the regions of origin were synovial fluid (n = 9; 24.3%), bone marrow (n = 6; 16.2%), adipose tissue (n = 3; 8.1%), mandibular condylar chondrocytes (n = 1; 2.7%), Wharton’s jelly (n = 1; 2.7%), and periodontal ligament (n = 1; 2.7%). After collection, these cells were differentiated into chondrocytes (n = 16; 43.2%), osteoblasts (n = 8; 21.6%), adipocytes (n = 7; 18.9%), neurons (n = 3; 8.1%), chondroblasts (n = 3; 8.1%), fibroblasts (n = 2; 5.4%), fibrochondrocytes (n = 2; 5.4%), osteoclasts (n = 1; 2.7%), macrophages (n = 1; 2.7%), and synoviocytes (n = 1; 2.7%). These studies aimed to treat osteoarthritis (n = 9; 24.3%), favor osteochondral neoformation/remodeling (n = 4; 10.8%), prevent fibrous ankylosis (n = 2; 5.4%), prevent subchondral bone resorption (n = 1; 2.7%), and treat anterior disc displacement (n = 1; 2.7%).

Analysis of in vivo studies (n = 57; 46.3%) showed that mesenchymal stem cells were collected from animals and humans (Table 2). Sources of animal origin were femoral bone (n = 6; 10.5%), tibia (n = 4; 7%), ilium (n = 3; 5.2%), TMJ subchondral bones (n = 1; 1.7%), synovial fluid (n = 1; 1.7%), condyle (n = 1; 1.7%) and glenoid fossa (n = 1; 1.7%). In turn, the sources of human origin were umbilical cord (n = 2; 3.5%), dental pulp (n = 1; 1.7%), condylar cartilage (n = 1; 1.7%) and adipose tissue (n = 1; 1.7%). Such studies aimed at treating osteoarthritis (n = 16; 28%), condylar cartilage defect (n = 5; 8.7%), joint ankylosis (n = 2; 3.5%), unilateral excision of the condyle (n = 1; 1.7%), subchondral bone deterioration (n = 1; 1.7%), hemimandible excision (n = 1; 1.7%), osteochondral defects (n = 1; 1.7%), excision condylar head (n = 1; 1.7%), hemifacial microsomia (n = 1; 1.7%), muscular or dental injuries such as anterior crossbite (n = 5; 8.7%), tension-responsive muscle hypertrophy mechanical (n = 2; 3.5%), lateral pterygoid hyperfunction (n = 1; 1.7%) and masseter myofascial pain (n = 1; 1.7%). In addition, age-related joint changes were also analyzed, such as postnatal condylar growth (n = 1; 1.7%), postnatal craniomandibular joint disc growth (n = 1; 1.7%), and condylar aging. (n = 1; 1.7%).

Among the results obtained in our study, expressions and inhibitions of biomarkers and their possible influence on different TMJ disorders were also analyzed (Table 3).

Among the clinical studies (n = 3; 2.4%), two were carried out in adults and one in children, with mesenchymal stem cells collected from the donor himself from regions of the spinal cord (n = 1), from adipose tissues (n = 1), and neurogenic synovial membranes (n = 1). The main objectives of these studies were to treat condylar hyperplasia (n = 1), internal TMJ dysfunction (n = 1), joint ankylosis, and first arch dysplasia syndrome (n = 1).

**Figure 6:**
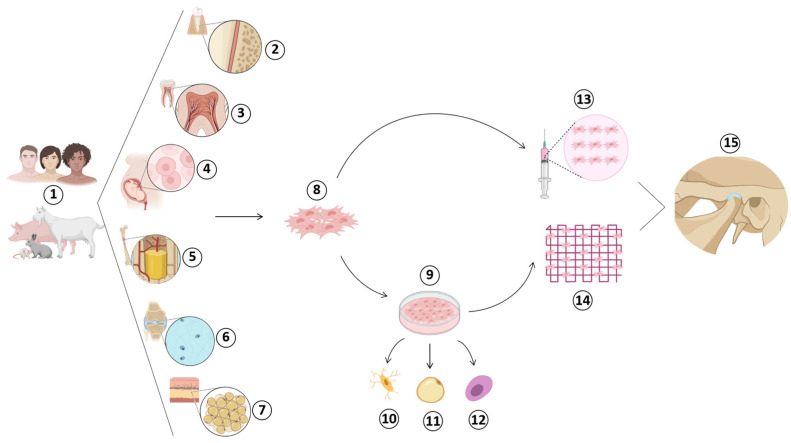
Stem cells scheme collection: source ①, donor area ②–⑦, differentiation ⑧, mode of application ⑬-⑭, and receptor region of mesenchymal stem cells ⑮. ①: Human or animal sources. ②: Periodontal ligament. ③: Dental pulp. ④: Umbilical cord. ⑤: Bone marrow. ⑥: Synovial fluid. ⑦: Adipose tissue. ⑧: Mesenchymal stem cell. ⑨: Cell culture and differentiation. ⑩: Osteocytes. ⑪: Adipocytes. ⑫: Chondrocytes. ⑬: Injection. ⑭ Scaffold. ⑮: Temporomandibular joint.

Figure 6 represents the collection scheme, according to findings from clinical studies, in vitro and in vivo studies, and donor tissues, differentiation, and applicability of mesenchymal stem cells.

## 4. Discussion

In recent years, studies indicated that using MSC is a promising strategy for treating TMD [47,60]. It is because they are multipotent cells that can be extracted from different sources such as bone marrow, the umbilical cord, muscle, adipose tissue, dermis, peripheral blood, liver, dental pulp, synovium, and synovial fluid of TMJ [16,61], in addition to being able to differentiate into other cell types, such as adipocytes, osteocytes, and chondrocytes [21]. Depending on the damage caused to the joint, stem cells can act in tissue repair and regeneration, suppressing the inflammatory process and modulating the immune system [17]. For example, in the initial stage of joint osteoarthritis, these cells have a protective, homeostatic, and regenerative function, while in the more advanced stages, they act by delaying tissue degeneration [62].

In our study, qualitative and quantitative analyses of the 125 selected articles on “TMD” and “stem cells” were carried out. In general, we observed that the clinical and experimental studies evaluated the action of mesenchymal stem cells collected from different sources and their possible reparative/regenerating potential against various TMJ disorders. In vivo and in vitro experimental studies were the main types found. The main donor sites were sources for bone marrow stroma, femoral bone, tibia, and synovial fluid. Chondrocytes and osteoblasts were the main cell types differed from stem cells for in vitro analysis. The central TMJ joint disorder investigated regarding the role of stem cells was osteoarthritis and, among muscle disorders, muscle hypertrophy. Joint modifications resulting from age, such as postnatal condylar growth and condylar aging, were also analyzed within the studies, as well as the evaluation of biomarkers, which, when expressed or inhibited, were associated—or not—with TMJ disorders.

The main database used in our analysis was WoS-CC. This was the main database chosen since it was designed to satisfy users in citation analysis and even provides graphic images of the analyzed results [63]. Scopus and Google Scholar databases were used to compare the number of citations. As already reported by other bibliometric studies [64,65], the database with the highest number of citations was Google Scholar. A possible explanation for this result may be related to the fact that Google Scholar presents citations of open-access books, thesis, dissertations, and online journals [65], journals that are not covered or not yet indexed by other databases, e-print archives, universities, or governmental and non-governmental organization web sites [66].

Although we selected all articles on the subject available at WoS-CC, regardless of whether they have citations, identifying studies with the highest number of citations is essential, as these data suggest that it gives it greater relevance in the field of knowledge [67]. Moreover, the most cited articles can be considered classic articles, exerting more significant scientific influence on the field and generating discussions about future research projections in the area [68]. However, it is worth mentioning that the time of publication can strongly influence the number of citations, since recently published studies do not have enough time to reach a large number of citations [69].

Analyzing articles published in WoS-CC on TMD and stem cells over the years brought some interesting findings. For example, even though the first article was published in 1992, only from 2008 onwards did the number of publications show regularity, with 2021 being the year with the highest number. In that year, in vivo studies were the majority (n = 8) and highlighted the ability to repair bone defects and TMJ tissue regeneration when treated with stem cells [39,41,42,53,70,71,72,73]. Such treatments were performed by injecting stem cells into the TMJ or using scaffolds enriched with them, thereby promoting guided tissue regeneration. A bibliometric study on artificial extracellular matrices showed a great trend in research on stem cells in tissue engineering and pointed out that in vitro studies are gradually being replaced by in vivo studies with a more clinical focus, justifying the increase in this type of research [74].

In general, when measured over the years, the type of study that most prevailed was once again in vivo (n = 51), followed by in vitro (n = 37, 30%). Such studies aimed at using mesenchymal stem cells to treat various joint disorders of the TMJ, mainly osteoarthritis, a disease characterized by chronic inflammation of the synovial tissue, as well as progressive cartilage degradation and remodeling of the subchondral bone [75]. Osteoarthritis is classified as articular TMD by the Diagnostic Criteria for Temporomandibular Disorders (DC/DTM) [76], an essential diagnostic tool used by clinical dentists and researchers. The prevalence of these studies contrasts with the epidemiological reality regarding TMD, since TMJ osteoarthritis is a highly prevalent disease [77].

The most published authors were Zhang M (n = 8; 6.4%) and Zhang J (n = 7; 5.6%). The two are part of the same team and share authorship in six articles whose central themes refer to the consequences and treatment of joint osteoarthritis. These were in vivo studies carried out with mice and published between 2014 and 2022. Interestingly, this was the period in which there were more publications on the subject, and the articles in which the authors shared authorship obtained a more significant number of quotes. As our study is about a specific theme, TMD and stem cells, when we compare our results with other bibliometric studies on each of these themes individually [23,24,25,26], the results are different, and several other authors appear. This is probably because the themes are very broad and cover classic studies, which have great impact, and were cited many times, serving as a basis and guideline to determine clinical conducts or even research protocols.

In the present study, the country that most published on the subject was China, which accounted for 61 published articles, reinforcing the result of other bibliometric studies on stem cells, in which China also ranked first with 1247 and 899 articles published, respectively [25,26]. Also, the USA obtained second place in these same studies, with 392 and 278 articles published, respectively. Our results indicate that despite having obtained second place in the number of publications on the subject, the USA had the most significant interaction with other countries. In a bibliometric study on TMD [24], the US leads in the number of articles published, with China in 15th place. Another study, also on TMD [23], again pointed to the USA being first place in the number of published articles and China in fourth place. A comparison between our results and the other cited bibliometric studies seems to indicate that China only leads studies on TMD specifically related to stem cells. 

Regarding keywords, they are used to facilitate the search for specific fields since the increase in the occurrence of one of these words can help to understand the search trend on a particular topic [12]. In our study, this analysis was performed using the density map, and it was observed that the most frequent words related to TMD were cartilage (n = 41; 4.42%), temporomandibular joint (n = 36; 3.88%), and osteoarthritis (n = 26; 2.80%). These words are closely related to each other, as osteoarthritis is a type of TMJ disorder characterized by progressive cartilage degeneration, abnormal subchondral bone remodeling, and synovitis [78]. Given the complexity of the disease, the most used conservative treatments are aimed at relieving symptoms [79]. However, the search for different treatments, such as those using stem cells with the aim of promoting tissue regeneration in the case of osteoarthritis, has recently grown [80], which may justify the search index on the field. Another bibliometric study on the performance of publications and research trends in TMD [81] found that the most frequent keywords were “orofacial pain”, “chronic pain”, “bruxism”, and “myofascial pain”. These words are related, respectively, to the classification of TMD as an orofacial and chronic pain; bruxism, which for years was confused with TMD, but is known to be different and may or may not be associated; and myofascial pain, which is one of the main types of muscular TMD.

Analyzing stem cells’ effects on TMJ tissues is crucial for understanding the formation and regeneration potential of such cell types on joint structures. Thus, it is worth highlighting the growing number of studies to describe the action of specific biomarkers in the natural development of healthy TMJ tissues or the presence of dysfunctions. In our study, the main biomarkers studied among the 125 articles were Ror2, TnC, Sox9, Protoglican4 (prr4) –null, TRPS1, Notch1, TNF-α, IFN-γ, Adrb2, HIF-1 alpha, GDF11, Ki67, FGF 18, Micro RNA-29b, Norepinephrine, Osteopontin, Type I Collagen, and Type II Collagen. Yang et al. [82] analyzing the Ror2 biomarker, found that its overexpression increases osteoclastogenic activity and subchondral bone loss, indicating that its presence is part of the formation process of bone defects in joint osteoarthritis. Other biomarkers investigated were Adrb2, whose increased expression induced subchondral bone loss in osteoarthritis [83], and Micro RNA-29b expression, which increases subchondral bone loss and osteoclast hyperfunction [84]. The analysis of the biomarkers HIF-1alpha, Ki67, FGF 18, collagen type I, and type II demonstrated that their expression causes cartilaginous regeneration through the induction and differentiation of chondrogenic cells. Furthermore, the inhibition of Notch1 promoted the temporary delay of the progress of cartilage degradation [37,72,74,85]. 

Although it was not selected among the 125 articles, it is worth mentioning an interesting recent article [86] that investigated the hypothesis that the polymorphism in the PAX7 gene would be associated with muscular TMD patients. As the authors explain, satellite cells (SC) are skeletal muscle stem cells, activated in cases of muscle injury. However, for them to be activated, growth factors must be present at the injury site, such as the expression of the transcription factor PAX7 in the differentiation of muscle cells. When they migrate to the site of injury, SCs also begin to express another myogenic regulatory factor, MyoD (myogenic differentiation). Thus, in the process of the differentiation of SC cells into myoblasts, there is an increase in MyoD expression and a reduction in PAX7 expression. Based on this principle, the authors selected two polymorphisms of a single nucleotide in the PAX7 gene (rs766325, rs6659735) and, as a result, they perceived that alterations in PAX7 can influence the muscle pathophysiology, and the homozygous genotype (GG) rs6659735 is apparently associated with the disorder muscle in individuals with TMD.

Another interesting point observed in the selected studies was that collecting stem cells was easy and the various properties found in these cells make them the target of the investigation. The source depends on the ability of stem cells to differentiate, the modulation of the immune and inflammatory response, and the power of paracrine communication [87]. In our study, the primary sources of stem cells were dental pulp, periodontal ligament, synovia, and synovial fluid. 

Mesenchymal stem cells derived from dental pulp are easily accessible, as they can be extracted from deciduous teeth naturally lost by children or third molars commonly indicated for extraction [88]. These cells have excellent differentiation potential and can be used to treat various tissues, including bones and cartilage [89]. Moreover, in dental-derived stem cells, NURR1 downregulation favors osteoblastic differentiation, with the further characteristic that these cells seem to possess an expression of stemness genes, such as the transcription factors Kruppel-like factor 4 (Klf-4), octamer-binding transcription factor 4 (Oct-4), homeobox transcription factor Nanog (Nanog), associated with the maintenance of self-renewal and multi-differentiation capacity and reported in MSCs of different origin, confirming the great potential of dental-derived stem cells in tissue regeneration [90,91]. Due to these characteristics, several studies have been developed to investigate these cells [92,93].

On the other hand, stem cells derived from the periodontal ligament are very potent cells that exhibit properties similar to those of mesenchymal stem cells derived from bone marrow, that is, they are clonogenic and have a high proliferative capacity and, specifically, are capable of regenerating periodontal tissues [94]. In addition, they exhibit the capacity for long-term survival, self-renewal, and show an ability to differentiate into specialized cells and regenerate various functional tissues [95,96].

Stem cells derived from the synovium have a high power of proliferation and chondrogenic differentiation. They can be extracted from tissue fragments removed in arthroscopy and can adhere to the cartilage when in a favorable environment [86]. Following, the first study on the characterization of cells derived from TMJ synovial fluid as stem cells was carried out by Koyama et al. [97]. In this, the authors confirmed that human synovial fluid is a good source of cells capable of differentiating into several other lineages, such as osteoblasts, chondrocytes, neurons, and adipocytes. Subsequently, Sun et al. [98] investigated both cells derived from fragments of synovia and mesenchymal stem cells derived from synovial fluid (SFCs) and identified that both SFCs can be induced to differentiate down osteogenic, chondrogenic, adipogenic, and neurogenic lineages in vitro and that probably, the intima is the most likely tissue origin of SFMSCs in the TMJ.

We also observed that some selected studies highlighted the benefits of an injection of stem cells into TMJs affected by some type of experimentally induced dysfunction and, for this purpose, each of these studies used different specific strategies, both to induce the lesion and to promote the injection of the cells. With the objective of evaluating in vitro and in vivo the effects of 20 minutes daily of low-intensity pulsed ultrasound (LIPUS) treatment, El-Bialy et al. [99] carried out a pilot study using bone marrow stromal cells (BMC) isolated from rabbits. These cells were expanded, differentiated into chondrogenic and osteogenic lineages, inserted into biodegradable scaffolds and later implanted into the amputated TMJ articular condylar defect. The results of this cited pilot study suggest that LIPUS can enhance chondrogenic and osteogenic differentiation of BMSCs in vitro as well as to enhance functional integration of these in vivo in rabbits. Zhang et al. [100] used a mouse model of TMJ osteoarthritis to weekly inject green fluorescent protein-labeled exogenous bone marrow stromal cells (GFP-BMSC) for 4, 8, and 12 successive weeks. They concluded that BMSCs were able to repair damaged cartilage by increasing matrix production and scavenging activity and by not increasing cell proliferation. Wang et al. [101] evaluated in vivo and in vitro the effects of an injection of bone marrow mesenchymal stem cell (BMSC)-derived small extracellular vesicles (BMSC-sEVs) on cartilage reconstruction with a TMJOA model in rabbits and confirmed that the BMSC-sEVs may play an important role in cartilage reconstruction in TMJOA via the autotaxin–YAP signaling axis. [49] also analyzed in vitro and in vivo the effects of an intra-articular injection of MSCs by placing these cells on the surface of TGFβ-loaded GelMA microspheres in a model of TMJ arthritis bilaterally induced in rats. They concluded that BMSCs-coated microspheres can effectively promote the repair and reconstruction of cartilage defects within the TMJ arthritis.

It is worth mentioning that among the selected articles, one tested the effectiveness of the intra-articular infiltration of autologous stem cells derived from adipose tissue in the upper compartment of the TMJ in four patients Carboni et al [102]. This study focused on verifying the results of this procedure regarding pain, joint noises, maximum mandibular opening, and mandibular movements. The authors concluded that the results of this preliminary study demonstrated that autologous stem cells derived from adipose tissue were able to resolve the symptoms presented by the patients, as well as being effective in restoring the structural anatomical integrity verified through the functional magnetic resonance imaging (MRI).

Thus, when analyzing all the results of our study, it is observed that the use of stem cells as a treatment strategy for TMD has been increasingly studied both in vitro and in vivo due to a certain ease of obtaining (depending on origin), the capacity for cell proliferation and differentiation and the regenerative potential of joint structures.

## 5. Conclusions

As far as we are concerned, this is the first bibliometric study that relates stem cells and TMD. With regard to the metrics, we observed that among the selected articles, although the first study was carried out in 1992, a more significant number of publications and citations occurred between the years 2012 and 2022. Furthermore, although 34 countries produce articles on the subject, China was in the lead, followed by the USA. The two authors with the highest number of published articles shared co-authorship in several studies. The main keywords found were “cartilage”, “temporomandibular joint”, “mesenchymal stem cells”, and “osteoarthritis”, which were closely related to the topic and indicated research trends in the area. When it comes to evaluating the knowledge produced, the selected articles evaluated cells extracted from different sources regarding their potential for differentiation and therapeutic intent. It was found that the most commonly used cell type were mesenchymal stem cells, which have a high proliferative capacity and a high power of differentiation into different cell types. In the selected studies, the main sources of these cells were the dental pulp, the periodontal ligament, the synovium, and the synovial fluid. In vivo studies were the most numerous, and this result possibly indicates a growing interest in using these cells to treat refractory patients with TMD. Based on our findings, it is suggested that future research in this area should be carried out in order to determine if there is any type and/or subtype of TMD that would benefit more from the treatment with stem cells, as well as if there would be any stage of development of TMD, i.e., more recent or more advanced, in which the use of stem cells would provide greater morphological and functional benefits to patients, consequently bringing benefits to their quality of life.

## Figures and Tables

**Table 1:** Main findings of in vitro studies regarding the origin of stem cells, differentiated cell types, and treatment purposes.

Characteristics of the In Vitro Studies Analyzed	Absolute Frequency	Relative Frequency (%)
**Source of origin of mesenchymal stem cells**		
**Animal origin**		
Bone marrow stroma	14	37.8
Fibrocartilage	1	2.7
Synovial fluid	1	2.7
Myelomonocytes	1	2.7
**Human origin**		
Synovial fluid	9	24.3
Bone marrow	6	16.2
Adipose tissue	3	8.1
Mandibular condylar chondrocytes	1	2.7
Wharton jam	1	2.7
Periodontal ligament	1	2.7
**Cell types differentiated from stem cells**		
Chondrocytes	16	43.2
Osteoblasts	8	21.6
Adipocytes	7	18.9
Neural	3	8.1
Chondroblasts	3	8.1
Fibroblasts	2	5.4
Fibrochondrocytes	2	5.4
Osteoclasts	1	2.7
Macrophages	1	2.7
**Purpose of TMD treatment**		
Osteoarthritis	8	21.6
Osteochondral Neoformation/remodeling	4	10.8
Fibrous ankylosis	2	5.4
Bone ankylosis	2	5.4
Subchondral bone resorption	1	2.7
Joint inflammation	1	2.7
Anterior disc displacement	1	2.7

**Table 2:** Main findings of in vivo studies regarding the source of stem cells and TMJ disorders to be treated.

In Vivo Studies Characteristics	Absolute Frequency	Relative Frequency (%)
**Source of origin of mesenchymal stem cells**		
Animal origin	6	10.5
Femoral bone	4	7
Tibia	3	5.2
Ilium	1	1.7
TMJ subchondral bones	1	1.7
Synovial fluid	1	1.7
Condyle	1	1.7
Glenoid fossa		
Human origin	2	3.5
The umbilical cord	1	1.7
Dental pulp	1	1.7
Condylar cartilage	1	1.7
**TMJ bone changes**		
Osteoarthritis	16	28
Condylar cartilage defect	5	8.7
Joint ankylosis	2	3.5
Unilateral excision of condyle	1	1.7
Subchondral bone deteriorationn	1	1.7
Hemimandible excision	1	1.7
Osteochondral defects	1	1.7
Condylar head excision	1	1.7
Hemifacial microsomia	1	1.7
**Malocclusions**		
Anterior crossbite	5	8.7
**Muscles disorders**		
Muscular hypertrofy	2	3.5
Lateral pterygoid hyperfunction	1	1.7
Masseter myofascial pain	1	1.7
**Age-related joint disorders**		
Condylar postnatal growth	1	1.7
Postnatal growth of Craniomandibular articular disk	1	1.7
Condylar aging	1	1.7

**Table 3:** The main biomarkers evaluated in selected studies, their expression or inhibition, and their possible influence on TMJ disorders.

Biomarkers	Expression/Inhibition	Relationship
Ror2	Expression	Induction of osteoclast formation
Tn-C	Expression	Chondrocyte formation
Sox9	Expression	Chondrocyte formation/cartilaginous regeneration
Proteoglycan 4 (Prg4)-null	Expression	Ectopic formation of mineralized tissues and osteophytes in the articular disc, mandibular condyle and glenoid fossa
TRPS1	Expression	Participates in ATM development
Notch1	Inhibition	Temporary delay in the progress of cartilage degradation
TNF-α	Expression	Inflammatory factor that delays improvement in osteoarthritis
IFN-γ	Expression	Inflammatory factor that delays improvement in osteoarthritis
Adrb2	Expression	Induces subchondral bone loss in osteoarthritis
HIF-1alfa	Expression	May induce stem cells to promote chondrogenic repair of condylar cartilage and inhibit bone sclerosis
GDF11	Expression	Inhibits chondrocyte adipogenesis
ki67	Expression	Cartilaginous regeneration
FGF 18	Expression	Cartilaginous regeneration
MicroRNA-29b	Expression	Increased subchondral bone loss and osteoclast hyperfunction
Norepinefrina	Expression	Degenerative changes of the condylar subchondral bone
Osteopontina	Expression	Induction in the differentiation of chondrogenic and osteogenic cells
Colágeno tipo I	Expression	Cartilaginous regeneration
Colágeno tipo II	Expression	Induction in the differentiation of chondrogenic and osteogenic cells

## Data Availability

Not applicable.

## Supplementary Materials

The following supporting information can be downloaded at: https://www.mdpi.com/article/10.3390/jfb14020103/s1. Supplementary file S1: The Search strategy and Supplementary file S2: Table S1—The 125 papers on the directions of Temporomandibular Disorders and Stem Cells.
