# Supplementary material for: Global Trends and Future Research Directions for Temporomandibular Disorders and Stem Cells
Source: J Funct Biomater. 2023 Feb 13;14(2):103. doi: 10.3390/jfb14020103 (PMC9965396; doi:10.3390/jfb14020103)
Supplement: Supplementary file 1 [file jfb-14-00103-s001.zip › Supplementary file 1.pdf]

Global Trends and Future Research Directions of Temporomandibular Disorders and Stem Cells

Zuleni Alexandre da Silva[1]; Wallacy Watson Pereira Melo[1]; Hadassa Helez Neves Ferreira[1]; Rafael Rodrigues Lima[1]; Renata Duarte de Souza-Rodrigues[1*].

Laboratory of Functional and Structural Biology, Institute of Biological Sciences, Federal University of Pará, Belém 66075-110, Brazil

* Correspondence: renataduarte@ufpa.br

Supplementary file 1: The Search strategy

TS= ("Cell, Stem" OR "Cells, Stem" OR "Stem Cell" OR "Progenitor Cells" OR "Cell, Progenitor" OR "Cells, Progenitor" OR "Progenitor Cell" OR "Mother Cells" OR "Cell, Mother" OR "Cells, Mother" OR "Mother Cell" OR "Colony-Forming Unit" OR "Colony Forming Unit" OR "Colony-Forming Units" OR "Colony Forming Units" OR "Adult Stem Cell" OR "Stem Cells, Adult" OR "Adult Somatic Stem Cells" OR "Somatic Adult Stem Cells" OR "Somatic Stem Cells" OR "Somatic Stem Cell" OR "Stem Cell, Somatic" OR "Stem Cells, Somatic" OR " IPS Cells" OR "Induced Pluripotent Stem Cell" OR "IPS Cell" OR "Cell, IPS" OR "Cells, IPS" OR "Human Induced Pluripotent Stem Cells" OR hiPSC OR "Human Induced Pluripotent Stem Cell" OR "Fibroblast-Derived Induced Pluripotent Stem Cells" OR "Fibroblast Derived Induced" OR "Pluripotent Stem Cells" OR "Fibroblast-Derived IPS Cells" OR "Cell, Fibroblast-Derived IPS" OR "Cells, Fibroblast-Derived IPS" OR "Fibroblast Derived IPS Cells" OR "Fibroblast-Derived IPS Cell" OR "IPS Cell, Fibroblast-Derived" OR "IPS Cells, Fibroblast-Derived" OR "Multipotent Stem Cells" OR "Multipotent Stem Cell" OR "Stem Cell, Multipotent" OR "Stem Cells, Multipotent" OR "Mesenchymal Stem Cells" OR "Stem Cell, Mesenchymal" OR "Stem Cells, Mesenchymal" OR "Mesenchymal Stem Cell" OR "Bone Marrow Mesenchymal Stem Cells" OR "Bone Marrow Mesenchymal Stem Cell" OR "Bone Marrow Stromal Cells" OR "Bone Marrow Stromal Cell" OR "Multipotent Bone Marrow Stromal Cell" OR "Multipotent Bone Marrow Stromal Cells" OR "Adipose-Derived Mesenchymal Stem Cells" OR "Adipose Derived Mesenchymal Stem Cells" OR "Adipose-Derived Mesenchymal Stromal Cells" OR "Adipose Derived Mesenchymal Stromal Cells" OR "Mesenchymal Stem Cells, Adipose-Derived" OR "Mesenchymal Stem Cells, Adipose Derived" OR "Adipose-Derived Mesenchymal Stem Cell" OR "Adipose Derived Mesenchymal Stem Cell" OR "Adipose Tissue-Derived Mesenchymal Stem Cell" OR "Adipose Tissue Derived Mesenchymal Stem Cell" OR "Adipose Tissue-Derived Mesenchymal Stem Cells" OR "Adipose Tissue Derived Mesenchymal Stem Cells" OR "Adipose Tissue-Derived Mesenchymal Stromal Cells" OR "Adipose Tissue Derived Mesenchymal Stromal Cells"

OR “Adipose Tissue-Derived Mesenchymal Stromal Cell” OR “Adipose Tissue Derived Mesenchymal Stromal Cell” OR “Mesenchymal Stromal Cells” OR “Mesenchymal Stromal Cell” OR “Stromal Cell, Mesenchymal” OR “Stromal Cells, Mesenchymal” OR “Multipotent Mesenchymal Stromal Cells” OR “Multipotent Mesenchymal Stromal Cell” OR “Mesenchymal Stromal Cells, Multipotent” OR “Mesenchymal Progenitor Cell” OR “Mesenchymal Progenitor Cells” OR “Progenitor Cell, Mesenchymal” OR “Progenitor Cells, Mesenchymal” OR “Wharton Jelly Cells” OR “Wharton's Jelly Cells” OR “Wharton's Jelly Cell” OR “Whartons Jelly Cells” OR “Bone Marrow Stromal Stem Cells” OR “Pluripotent Stem Cells” OR “Pluripotent Stem Cell” OR “Stem Cell, Pluripotent” OR “Stem Cells, Pluripotent” OR “Induced Pluripotent Stem Cells” OR “IPS Cells” OR “Induced Pluripotent Stem Cell” OR “IPS Cell” OR “Cell, IPS” OR “Cells, IPS” OR “Human Induced Pluripotent Stem Cells” OR hiPSC OR “Human Induced Pluripotent Stem Cell” OR “Fibroblast-Derived Induced Pluripotent Stem Cells” OR “Fibroblast Derived Induced Pluripotent Stem Cells” OR “Fibroblast-Derived IPS Cells” OR “Cell, Fibroblast-Derived IPS” OR “Cells, Fibroblast-Derived IPS” OR “Fibroblast Derived IPS Cells” OR “Fibroblast-Derived IPS Cell” OR “IPS Cell, Fibroblast-Derived” OR “IPS Cells, Fibroblast-Derived” OR “Temporomandibular joint derived synovial stem cells” OR “Fibrocartilage stem cells” OR “TMJA bone marrow stem cells” OR “effect of bone marrow-derived stem cells”) AND TS= (“Temporomandibular joint disorders” OR “Disorder, Temporomandibular Joint” OR “Disorders, Temporomandibular Joint” OR “Joint Disorder, Temporomandibular” OR “Joint Disorders, Temporomandibular” OR “Temporomandibular Joint Disorder” OR “TMJ Disorders” OR “Disorder, TMJ” OR “Disorders, TMJ” OR “TMJ Disorder” OR “Temporomandibular Disorders” OR “Disorder, Temporomandibular” OR “Disorders, Temporomandibular” OR “Temporomandibular Disorder” OR “Temporomandibular Joint Diseases” OR “Diseases, Temporomandibular Joint" OR "Joint Disease, Temporomandibular" OR "Joint Diseases, Temporomandibular" OR "Temporomandibular Joint Disease" OR "TMJ Diseases" OR "Disease, TMJ" OR "Diseases, TMJ" OR "TMJ Disease" OR "Temporomandibular Joint Dysfunction Syndrome" OR "Myofascial Pain Dysfunction Syndrome, Temporomandibular Joint" OR "TMJ Syndrome" OR "Syndrome, TMJ" OR "Costen's Syndrome" OR "Costen Syndrome" OR "Costens Syndrome" OR "Syndrome, Costen's" OR "Temporomandibular Joint Syndrome" OR "Joint Syndrome, Temporomandibular" OR "Syndrome, Temporomandibular Joint" OR "Craniomandibular Disorders" OR "Craniomandibular Disorder" OR "Disorder, Craniomandibular" OR "Disorders, Craniomandibular" OR "Craniomandibular Diseases" OR "Craniomandibular Disease" OR "Disease, Craniomandibular" OR "Diseases, Craniomandibular" OR "Myofascial Pain Syndromes" OR "Myofascial Pain Syndrome" OR "Pain Syndrome, Myofascial" OR "Pain Syndromes, Myofascial" OR "Syndrome, Myofascial Pain" OR "Syndromes, Myofascial Pain" OR "Myofascial Trigger Point Pain" OR "Trigger Point Pain, Myofascial" OR "TMJ Osteoarthritis" OR "Temporomandibular joint osteoarthritis" OR "TMJ Osteoarthrosis" OR "Temporomandibular joint osteoarthrosis" OR "Internal derangements of temporomandibular joint" OR "Internal derangements of the temporomandibular joint" OR "Internal derangements of TMJ" OR "TMJ Internal derangements" OR "Anterior disc displacement of temporomandibular joint" OR "Disc displacement of the temporomandibular joint" OR "Anterior disc displacement the temporomandibular joint" OR "Temporomandibular joint disc displacement with

reduction" OR "TMJ disc displacement with reduction" OR "Disc displacement reduction of the temporomandibular joint " OR "Temporomandibular joint disc displacement without reduction" OR "TMJ disc displacement without reduction" OR "Reducing disc displacement of the temporomandibular joint" OR "Non reducing disc displacement of the temporomandibular joint" OR "Temporomandibular joint" OR "Joint, Temporomandibular" OR "Joints, Temporomandibular" OR "Temporomandibular Joints" OR TMJ OR “Temporomandibular SMSCs”).
