# Supplementary material for: Global Trends and Future Research Directions for Temporomandibular Disorders and Stem Cells
Source: J Funct Biomater. 2023 Feb 13;14(2):103. doi: 10.3390/jfb14020103 (PMC9965396; doi:10.3390/jfb14020103)
Supplement: Supplementary file 1 [file jfb-14-00103-s001.zip › Supplementary file 2.pdf]

Global Trends and Future Research Directions of Temporomandibular Disorders and Stem Cells

Zuleni Alexandre da Silva[1]; Wallacy Watson Pereira Melo[1]; Hadassa Helez Neves Ferreira[1]; Rafael Rodrigues Lima[1]; Renata Duarte de Souza-Rodrigues[1*].

Laboratory of Functional and Structural Biology, Institute of Biological Sciences, Federal University of Pará, Belém 66075-110, Brazil

* Correspondence: renataduarte@ufpa.br

Supplementary file 1: The Search strategy

TS= ("Cell, Stem" OR "Cells, Stem" OR "Stem Cell" OR "Progenitor Cells" OR "Cell, Progenitor" OR "Cells, Progenitor" OR "Progenitor Cell" OR "Mother Cells" OR "Cell, Mother" OR "Cells, Mother" OR "Mother Cell" OR "Colony-Forming Unit" OR "Colony Forming Unit" OR "Colony-Forming Units" OR "Colony Forming Units" OR "Adult Stem Cell" OR "Stem Cells, Adult" OR "Adult Somatic Stem Cells" OR "Somatic Adult Stem Cells" OR "Somatic Stem Cells" OR "Somatic Stem Cell" OR "Stem Cell, Somatic" OR "Stem Cells, Somatic" OR " IPS Cells" OR "Induced Pluripotent Stem Cell" OR "IPS Cell" OR "Cell, IPS" OR "Cells, IPS" OR "Human Induced Pluripotent Stem Cells" OR hiPSC OR "Human Induced Pluripotent Stem Cell" OR "Fibroblast-Derived Induced Pluripotent Stem Cells" OR "Fibroblast Derived Induced" OR "Pluripotent Stem Cells" OR "Fibroblast-Derived IPS Cells" OR "Cell, Fibroblast-Derived IPS" OR "Cells, Fibroblast-Derived IPS" OR "Fibroblast Derived IPS Cells" OR "Fibroblast-Derived IPS Cell" OR "IPS Cell, Fibroblast-Derived" OR "IPS Cells, Fibroblast-Derived" OR "Multipotent Stem Cells" OR "Multipotent Stem Cell" OR "Stem Cell, Multipotent" OR "Stem Cells, Multipotent" OR "Mesenchymal Stem Cells" OR "Stem Cell, Mesenchymal" OR "Stem Cells, Mesenchymal" OR "Mesenchymal Stem Cell" OR "Bone Marrow Mesenchymal Stem Cells" OR "Bone Marrow Mesenchymal Stem Cell" OR "Bone Marrow Stromal

Cells” OR “Bone Marrow Stromal Cell” OR “Multipotent Bone Marrow Stromal Cell” OR “Multipotent Bone Marrow Stromal Cells” OR “Adipose-Derived Mesenchymal Stem Cells” OR “Adipose Derived Mesenchymal Stem Cells” OR “Adipose-Derived Mesenchymal Stromal Cells” OR “Adipose Derived Mesenchymal Stromal Cells” OR “Mesenchymal Stem Cells, Adipose-Derived” OR “Mesenchymal Stem Cells, Adipose Derived” OR “Adipose-Derived Mesenchymal Stem Cell” OR “Adipose Derived Mesenchymal Stem Cell” OR “Adipose Tissue-Derived Mesenchymal Stem Cell” OR “Adipose Tissue Derived Mesenchymal Stem Cell” OR “Adipose Tissue-Derived Mesenchymal Stem Cells” OR “Adipose Tissue Derived Mesenchymal Stem Cells” OR “Adipose Tissue-Derived Mesenchymal Stromal Cells” OR “Adipose Tissue Derived Mesenchymal Stromal Cells” OR “Adipose Tissue-Derived Mesenchymal Stromal Cell” OR “Adipose Tissue Derived Mesenchymal Stromal Cell” OR “Mesenchymal Stromal Cells” OR “Mesenchymal Stromal Cell” OR “Stromal Cell, Mesenchymal” OR “Stromal Cells, Mesenchymal” OR “Multipotent Mesenchymal Stromal Cells” OR “Multipotent Mesenchymal Stromal Cell” OR “Mesenchymal Stromal Cells, Multipotent” OR “Mesenchymal Progenitor Cell” OR “Mesenchymal Progenitor Cells” OR “Progenitor Cell, Mesenchymal” OR “Progenitor Cells, Mesenchymal” OR “Wharton Jelly Cells” OR “Wharton's Jelly Cells” OR “Wharton's Jelly Cell” OR “Whartons Jelly Cells” OR “Bone Marrow Stromal Stem Cells” OR “Pluripotent Stem Cells” OR “Pluripotent Stem Cell” OR “Stem Cell, Pluripotent” OR “Stem Cells, Pluripotent” OR “Induced Pluripotent Stem Cells” OR “IPS Cells” OR “Induced Pluripotent Stem Cell” OR “IPS Cell” OR “Cell, IPS” OR “Cells, IPS” OR “Human Induced Pluripotent Stem Cells” OR hiPSC OR “Human Induced Pluripotent Stem Cell” OR “Fibroblast-Derived Induced Pluripotent Stem Cells” OR “Fibroblast Derived Induced Pluripotent Stem Cells” OR “Fibroblast-Derived IPS Cells” OR “Cell, Fibroblast-Derived IPS” OR “Cells, Fibroblast-Derived IPS” OR “Fibroblast Derived IPS Cells” OR “Fibroblast-Derived IPS Cell” OR “IPS Cell, Fibroblast-Derived” OR “IPS Cells, Fibroblast-Derived” OR “Temporomandibular joint derived synovial stem cells” OR “Fibrocartilage stem cells” OR “TMJA bone marrow stem cells” OR “effect of bone marrow-derived stem cells”) AND TS= (“Temporomandibular joint disorders” OR “Disorder, Temporomandibular Joint” OR “Disorders, Temporomandibular Joint” OR “Joint Disorder, Temporomandibular” OR “Joint Disorders, Temporomandibular” OR “Temporomandibular Joint Disorder” OR “TMJ Disorders” OR “Disorder, TMJ” OR “Disorders, TMJ” OR “TMJ Disorder” OR “Temporomandibular Disorders” OR “Disorder, Temporomandibular” OR “Disorders, Temporomandibular” OR “Temporomandibular Disorder” OR “Temporomandibular Joint Diseases” OR “Diseases, Temporomandibular Joint" OR "Joint Disease, Temporomandibular" OR "Joint Diseases, Temporomandibular" OR "Temporomandibular Joint Disease" OR "TMJ Diseases" OR "Disease, TMJ" OR "Diseases, TMJ" OR "TMJ Disease" OR "Temporomandibular Joint Dysfunction Syndrome" OR "Myofascial Pain Dysfunction Syndrome, Temporomandibular Joint" OR "TMJ Syndrome" OR "Syndrome, TMJ" OR "Costen's Syndrome" OR "Costen Syndrome" OR "Costens Syndrome" OR "Syndrome, Costen's" OR "Temporomandibular Joint Syndrome" OR "Joint Syndrome, Temporomandibular" OR "Syndrome, Temporomandibular Joint" OR "Craniomandibular Disorders" OR "Craniomandibular Disorder" OR "Disorder, Craniomandibular" OR "Disorders, Craniomandibular" OR "Craniomandibular Diseases" OR "Craniomandibular Disease" OR "Disease, Craniomandibular" OR "Diseases, Craniomandibular" OR "Myofascial Pain Syndromes" OR "Myofascial Pain Syndrome" OR "Pain Syndrome, Myofascial" OR "Pain Syndromes, Myofascial" OR "Syndrome, Myofascial Pain" OR "Syndromes, Myofascial

Pain" OR "Myofascial Trigger Point Pain" OR "Trigger Point Pain, Myofascial" OR "TMJ Osteoarthritis" OR "Temporomandibular joint osteoarthritis" OR "TMJ Osteoarthrosis" OR "Temporomandibular joint osteoarthrosis" OR "Internal derangements of temporomandibular joint" OR "Internal derangements of the temporomandibular joint" OR "Internal derangements of TMJ" OR "TMJ Internal derangements" OR "Anterior disc displacement of temporomandibular joint" OR "Disc displacement of the temporomandibular joint" OR "Anterior disc displacement the temporomandibular joint" OR "Temporomandibular joint disc displacement with reduction" OR "TMJ disc displacement with reduction" OR "Disc displacement reduction of the temporomandibular joint " OR "Temporomandibular joint disc displacement without reduction" OR "TMJ disc displacement without reduction" OR "Reducing disc displacement of the temporomandibular joint" OR "Non reducing disc displacement of the temporomandibular joint" OR "Temporomandibular joint" OR "Joint, Temporomandibular" OR "Joints, Temporomandibular" OR "Temporomandibular Joints" OR TMJ OR "Temporomandibular SMSCs").

Global Trends and Future Research Directions of Temporomandibular Disorders and Stem Cells

Zuleni Alexandre da Silva[1]; Wallacy Watson Pereira Melo[1]; Hadassa Helez Neves Ferreira[1]; Rafael Rodrigues Lima[1]; Renata Duarte de Souza-Rodrigues[1*].

Laboratory of Functional and Structural Biology, Institute of Biological Sciences, Federal University of Pará, Belém 66075-110, Brazil

* Correspondence: renataduarte@ufpa.br

Table S1. The 125 papers on the directions of Temporomandibular Disorders and Stem Cells.

Rank	Article title	Authors	Times cited (Citation average)			DOI	*Study design*	Abstract
			WoS-CC	Scopus	Google scholar			
**1**	The relationship of undifferentiated mesenchymal cells to TMJ articular tissue thickness	Bibb, CA. et al., 1992	22 (0.73)	23 (0.77)	43 (1.43)	10.1177/0022034592071011 1001	*In vitro*	Evaluated the assessed of the strength of the correlation between the presence of undifferentiated mesenchymal cells and soft tissue thickness in adult TMJs and suggests that other biological processes be studied to assess this relationship
**2**	Effects of lateral pterygoid muscle hyperactivity on differentiation of mandibular condyles in rats	Takahashi, I. et al., 1995	25 (0.93)	31 (1.15)	42 (1.56)	10.1002/ar.10 92410306	*In vivo*	Demonstrated the role of the masseter muscle as a strong influence on the differentiation of progenitor cells in TMJ
**3**	Engineered cartilage, bone, joints, and menisci - Potential for temporomandibular joint reconstruction	Glowacki, J. 2001	21 (1)	26 (1,24)	50 (2,38)	10.1159/0000 47895	Literature review	Therapies based on tissue engineering for tissue regeneration were analyzed
**4**	Mechanical strain leads to condylar growth in adult rats	Xiong, H. et al., 2005	20 (1.18)	22 (1.29)	45 (2.65)	10.2741/1507	*In vivo*	Evaluated the rate of mesenchymal cell proliferation in mandibular condyles to correlate with the expression of SOX9 and type II collagen under mechanical stress
**5**	High-dose Chemotherapy with Autologous Stem Cell Rescue in Stage IIIB Inflammatory Breast Cancer	Yalamanchili, K. et al., 2008	2(0.14)	5(0.36)	9(0.64)		Clinical trial study	Patients undergoing transplantation with autologous stem cells were evaluated for the effectiveness of high-dose chemotherapy

**6**	Differential gene expression in the perichondrium and cartilage of the neonatal mouse temporomandibular joint	Hinton, RJ. et al., 2009	25 (1.92)	29 (2.23)	45 (3.46)	10.1111/j.1601-6343.2009.01450.x	*In vitro*	Investigated the expression of genes in the perichondrium of the condylar cartilage to guide new studies for tissue regeneration
**7**	Functional alterations in mechanical loading of condylar cartilage induces changes in the bony subcondylar region	Papachristou, DJ. et al., 2009	16(1.23)	17(1.31)	31(2.38)	10.1016/j.archoralbio.2009.08.010	*In vivo*	The JNK/ERK-AP-1/Runx2 signaling axis was investigated to verify the extent to which mechanotransduction events in chondrocytes are "detected" in the subchondral bone area under altered functional load
**8**	Use of synovium-derived stromal cells and chitosan/collagen type I scaffolds for cartilage tissue engineering	Gong, ZC. et al., 2010	25 (2.08)	30 (2.50)	42 (3.50)	10.1088/1748-6041/5/5/055005	*In vitro/In vivo*	Investigated the action of synovial stromal cells in chitosan/collagen type I (CS/COL-I) scaffolds for cartilage neoformation
**9**	Temporomandibular Joint Reconstruction: from Alloplastic Prosthesis to Bioengineering Tissue	Yuan, K. et al., 2010	16(1.33)	15(1.25)	24(2.00)		Literature review	Discussed the pathogenesis of TMD, as well as traditional treatments and within tissue bioengineering
**10**	Postnatal Development of Type II Collagen and Aggrecan mRNA Expression in a Rabbit Craniomandibular Joint	Feng, JY. et al., 2010	2(0.17)	3(0.25)	7(0.58)	10.1002/ar.21200	*In vivo*	Studied growth changes in the condyle and TMJ joint, as well as the process of chondrocyte differentiation

**11**	Postnatal Development of Type II Collagen and Aggrecan mRNA Expression in a Rabbit Craniomandibular Joint	Feng, JY. et al., 2010	2(0.17)	3(0.25)	7(0.58)	10.1002/ar.21200	*In vivo*	Studied growth changes in the condyle and TMJ joint, as well as the process of chondrocyte differentiation
**12**	Development of temporomandibular joint ankylosis in rats using stem cells and bone graft	Porto, GG. et al., 2011	6(0.55)	7(0.64)	11(1.00)	10.1016/j.ijom.2011.07.910	*In vivo*	Evaluated a model study to induce bone ankylosis of the TMJ in rats through bone grafting or stem cell placement
**13**	The primordium of a biological joint replacement: Coupling of two stem cell pathways in biphasic ultrarapid compressed gel niches	Brady, MA. et al., 2011	19(1.73)	22(2.00)	29(2.64)	10.1016/j.jcms.2010.07.002	*In vitro*	Analyzed a single-source biomaterial and mesenchymal stem cells to design an *in vitro* biphasic osteochondral construct for future *in vivo* implantation
**14**	Differentiation of temporomandibular joint synovial mesenchymal stem cells into neuronal cells in vitro: an in vitro study	Liu, ZM. et al., 2011	9(0.82)	9(0.82)	15(1.36)	10.1042/CBI20100144	*In vitro*	Investigated the ability of synovial mesenchymal stem cells to differentiate into neuronal tissues through induction by basic fibroblast growth factor
**15**	Basic fibroblast growth factor enhances osteogenic and chondrogenic differentiation of human bone marrow mesenchymal stem cells in coral scaffold constructs	Zheng, YH. et al., 2011	22 (2.00)	24 (2.18)	54 (4.91)	10.1002/term.346	*In vitro*	Evaluated the feasibility of using bone marrow mesenchymal stem cells in scaffolds for construction of the mandibular condyle

**16**	Cell-based Meniscal Tissue Engineering: A Case for Synoviocytes.	Fox, DB. Et al., 2011	16(1.45)	18(1.64)	30(2.73)	10.1007/s11999-011-1824-z	Literature review	Evaluated available treatments for miniscal conditions and how cells of synovial origin can be used for this treatment
**17**	RhoA/Rho kinase signaling regulates transforming growth factor-beta 1-induced chondrogenesis and actin organization of synovium-derived mesenchymal stem cells through interaction with the Smad pathway	Xu, T. et al., 2012	37 (3.70)	Não localizado	67 (6.70)	10.3892/ijmm.2012.1107	*In vitro*	Evaluated the activation of the RhoA/Rho kinase pathway and its contribution to chondrogenesis and organization of the actin cytoskeleton of TMJ synovial mesenchymal stem cells in response to transforming growth factor-β1 (TGF-β1)
**18**	Trps1 is necessary for normal temporomandibular joint development	Michikami, I. et al., 2012	19(1.90)	20(2.00)	27(2.70)	10.1007/s00441-012-1372-1	*In vivo*	Analyzed the pattern expression of Trps1 during the development of the TMJ, which proved to be essential in the development of the normal condyle
**19**	Treatment of Irradiated Mandibles With Mesenchymal Stem Cells Transfected With Bone Morphogenetic Protein 2/7	Zhang, WB. et al., 2012	15(1.50)	18(1.80)	28(2.80)	10.1016/j.joms.2012.01.022	*In vivo*	Evaluated whether mesenchymal stem cells transfected with bone morphogenetic protein could increase bone remodeling after radiotherapy

**20**	Morphological effects of mesenchymal stem cells and pulsed ultrasound on condylar growth in rats: a pilot study	Oyonarte, R. et al., 2013	10(1.11)	11(1.22)	16(1.78)		*In vivo*	Evaluated by the through computer omography the morphological effect of intra-articular injection of mesenchymal stem cells and/or low intensity pulsed ultrasound in mandibular condyles
**21**	Mesenchymal stem cells and platelet gel improve bone deposition within CAD-CAM custom-made ceramic HA scaffolds for Condyle Substitution	Ciocca, L. et al., 2013	19 (2.11)	22 (2.44)	36 (4.00)	10.1155/2013/ 549762	*In vivo*	Promoted regenerative approach using mesenchymal stem cells aspirated from the iliac crest, added in pure and porous hydroxyapatite scaffolds to replace the TMJ condyle
**22**	Biological Reconstruction of the Temporomandibular Joint by Chondro-Osseous Graft: Clinical and Experimental Study	Kummoona, RK, 2013	4(0.44)	4(0.44)	12(1.33)	10.1097/SCS. 0b013e318258 8116	Clinical trial study	Reconstruction of the mandibular condyle with chondro-osseous graft performed in children
**23**	Decreased osteogenesis in stromal cells from radiolucent zone of human TMJ ankylosis	Xiao, E. et al., 2013	21 (2.33)	21 (2.33)	24 (2.67)	10.1177/0022 034513483471	*In vitro*	Investigated the presence of multipotent cells in the radiolucent zone, proving the existence of cells similar to mesenchymal cells and evaluating their proliferative and osteogenic capacity

**24**	Periodontal Ligament Mesenchymal Stromal Cells Increase Proliferation and Glycosaminoglycans Formation of Temporomandibular Joint Derived Fibrochondrocytes	Zhang, JL. et al., 2014	2(0.25)	5(0.63)	10(1.25)	10.1155/2014/410167	*In vitro*	Evaluated the possibility of fibrocartilage regeneration with a mixture of TMJ fibrochondrocytes and mesenchymal stem cells derived from the periodontal ligament
**25**	The Pilot Study of Fibrin with Temporomandibular Joint Derived Synovial Stem Cells in Repairing TMJ Disc Perforation	Wu, Y. et al., 2014	67 (8.38)	35 (4.38)	61 (7.63)	10.1155/2014/454021	*In vitro/ in vivo*	Evaluated the repair of TMJ disc perforation using hybrid fibrin/chitosan scaffold combined with synovial mesenchymal stem cells
**26**	Mutual effect between neuropeptides and inflammatory cytokines in neurogenic SMSCs of human temporomandibular joint	Liu, ZM. et al., 2014	4(0.50)	4(0.50)	7(0.88)	10.1007/s11596-014-1323-z	*In vitro*	Examined the ability of substance P (SP) and peptide to stimulate synovial and neurogenic mesenchymal stem cells, evaluated the mutual effects of inflammatory cytokines and neuropeptides, and the analgesic effect of hyaluronic acid
**27**	Decreased bone marrow stromal cells activity involves in unilateral anterior crossbite-induced early subchondral bone loss of temporomandibular joints	Yang, T. et al., 2014	15(1.88)	14(1.75)	15(1.88)	10.1016/j.archoralbio.2014.05.024	*In vivo*	Evaluates the changes in osteoclast and osteoblast activity in the subchondral bone of the TMJ in a unilateral crossbite model

**28**	Synovium Fragment-Derived Cells Exhibit Characteristics Similar to Those of Dissociated Multipotent Cells in Synovial Fluid of the Temporomandibular Joint	Sun, YP. et al., 2014	19 (2.38)	27 (3.38)	31 (3.88)	10.1371/journal.pone.0101896	*In vitro*	Characterized synovial fluid stem cells to understand their role in TMJ
**29**	Overexpression of Indian hedgehog partially rescues short stature homeobox 2-overexpression-associated congenital dysplasia of the temporomandibular joint in mice	Li, XH. et al., 2015	2(0.29)	2(0.29)	7(1.00)	10.3892/mmr.2015.3959	*In vivo*	Investigated whether Ihh expression can rescue Sox2 overexpression causing congenital TMJ dysplasia
**30**	Subchondral bone changes and chondrogenic capacity of progenitor cells from subchondral bone in the collagenase-induced temporomandibular joints osteoarthritis rabbit model	Wu, GM. et al., 2015	5(0.71)	4(0.57)	7(1.00)		*In vivo*	Characterized the subchondral bone changes and biological activity of subchondral bone progenitor cell populations in TMJ osteoarthritis
**31**	Engineered microporosity: enhancing the early regenerative potential of decellularized temporomandibular joint discs	Juran, CM. et al., 2015	23 (3.29)	26 (3.71)	34 (4.86)	10.1089/ten.tea.2014.0250	*In vitro*	Evaluated the effectiveness of ex vivo laser micropatterning (LMP) derived TMJ disk scaffolds to improve cell integration

**32**	Stem Cells for Temporomandibular Joint Repair and Regeneration	Zhang, SP. et al., 2015	25 (3.57)	27 (3.86)	44 (6.29)	10.1007/s12015-015-9604-x	Literature review	Addressed the most recent advances in the development of stem cell treatments for TMJ
**33**	Norepinephrine Regulates Condylar Bone Loss via Comorbid Factors	Jiao, K. et al., 2015	15(2.14)	16(2.29)	17(2.43)	10.1177/0022034515577677	*In vivo*	Evaluated the gene expression parameters in bone and cartilaginous remodeling in a murine model
**34**	Osteoclast Deficiency Contributes to Temporomandibular Joint Ankylosed Bone Mass Formation	He, LH. et al., 2015	9(1.29)	12(1.71)	17(2.43)	10.1177/0022034515599149	*In vitro*	Characterized osteogenesis of bone marrow stem cells and bone marrow myelocytes and evaluated TMJ bone density
**35**	Wnt5a/Ror2 Mediates Temporomandibular Joint Subchondral Bone Remodeling	Yang, T. et al., 2015	25 (3.57)	28 (4.00)	37 (5.29)	10.1177/0022034515576051	*In vivo*	Analyzed the action of Wnt5a/Ror2 signaling derived from bone marrow stromal stem cells in regulating the migration and differentiation of osteoclast precursors
**36**	Osteophyte formation and matrix mineralization in a TMJ osteoarthritis mouse model are associated with ectopic hedgehog signaling	Bechtold, TE. et al., 2016	23 (3.83)	21 (3.50)	29 (4.83)	10.1016/j.matbio.2016.03.001	*In vivo*	Investigated the main stages of osteophyte formation in the glenoid eminence bos the hypothesis that the Ihh signaling pathway may be involved in this process

**37**	Upregulation of proangiogenic factors expression in the synovium of temporomandibular joint condylar hyperplasia	Guo, HL. et al., 2016	5(0.83)	5(0.83)	7(1.17)	10.1016/j.oooo.2015.11.004	*In vitro*	Investigated the expression of angiogenic-associated factors in the synovium and discussed their role in the progression of condylar hyperplasia
**38**	Modeling Mesenchymal Stem Cells in TMJ Rheumatoid Arthritis and Osteoarthritis Therapy	Serakinci, N. et al., 2017	8(1.60)	10(2.00)	11(2.20)	10.1615/CritRevEukaryotGeneExpr.2017019380	Literature review	Discussed the use of human mesenchymal stem cells in the treatment of inflammatory diseases of the TMJ
**39**	Acquired heterotopic ossification of the temporomandibular joint	Mercuri, LG. e Saltzman, BM, 2017	18(3.60)	24(4.80)	29(5.80)	10.1016/j.ijom.2017.06.016	Literature review	Etiology, diagnosis and treatment of heterotropic TMJ ossification
**40**	Hydroxyapatite collagen scaffold with autologous bone marrow aspirate for mandibular condylar reconstruction	Howlader, D. et al., 2017	10(2.00)	12(2.40)	17(3.40)	10.1016/j.jcms.2017.06.022	Clinical trial study	Evaluated the efficiency of bio-scaffold with bone marrow stem cell aspirate in the reconstruction of mandibular condyle with ankylosis
**41**	Isolation and characterization of mesenchymal stromal progenitors from the temporomandibular joint disc	Lavi, A. et al., 2017	0	2(0.40)	7(1.40)	10.1002/term.2055	*In vivo*	Under the hypothesis that the TMJ disc contains multipotent stromal progenitor cells that may play an important role in disc regeneration this study isolated and characterized TMJ progenitor cells

**42**	Mesenchymal Stem Cells for Cartilage Regeneration of TMJ Osteoarthritis	Cui, DX. et al., 2017	26 (5.20)	37 (7.40)	54 (10.80)	10.1155/2017/5979741	Literature review	Addressed the pathogenesis of cartilage degeneration in TMJ osteoarthritis and new cartilage regeneration approaches, emphasizing potential sources of mesenchymal stem cells
**43**	A review of in-vitro fibrocartilage tissue engineered therapies with a focus on the temporomandibular joint	Lowe, J. et al., 2017	21 (4.20)	23 (4.60)	30 (6.00)	10.1016/j.archoralbio.2017.07.013	Literature review	Demonstrated the progress of TMJ in vitro tissue engineering initiatives and compared to more advanced fibrocartilage fields such as the annulus fibrosus of invertebrate discs and the meniscus of the knee
**44**	Matrix replenishing by BMSCs is beneficial for osteoarthritic temporomandibular joint cartilage	Zhang, M. et al., 2017	22 (4.40)	23 (4.60)	29 (5.80)	10.1016/j.joca.2017.05.007	*In vitro*	Evaluated the matrix renewal as the main requirement for cartilage regeneration in TMJ joint osteoarthritis
**45**	Impact of Autologous Bone Marrow-Derived Stem Cells on Degenerative Changes of Articulating Surfaces Associated With the Arthritic Temporomandibular Joint: An Experimental Study in Rabbits	Zaki, AA. et al., 2017	4(0.80)	4(0.80)	11(2.20)	10.1016/j.joms.2017.05.001	*In vivo*	Evaluated the effect of bone marrow-derived stem cells on TMJ degenerative changes associated with induced arthritis

**46**	The LncRNA ZBED3-AS1 induces chondrogenesis of human synovial fluid mesenchymal stem cells	Ou, FR. et al., 2017	25 (5.00)	26 (5.20)	30 (6.00)	10.1016/j.bbrc .2017.04.090	*In vitro*	Examined the expression of ZBED3-AS1 and zbed3 in induced synovial mesenchymal stem cells, to detect its role in chondrogenesis and examined its relationship with zbed3 and Wnt signaling
**47**	Roles of Ihh signaling in chondroprogenitor function in postnatal condylar cartilage	Kurio, N. et al., 2018	23 (5.75)	25 (6.25)	29 (7.25)	10.1016/j.mat bio.2018.02.0 11	*In vivo*	Investigated the cellular organization of the polymorphic/progenitor layer, purpose and function of chondroprogenitor cells, and the roles of Ihh signaling in these processes
**48**	Co-culture of bone marrow stromal cells and chondrocytes in vivo for the repair of the goat condylar cartilage defects	Sun, H. et al., 2018	6(1.50)	7(1.75)	12(3.00)	10.3892/etm.2 018.6551	*In vivo*	Explored the feasibility of differentiating of bone marrow stromal cells into chondrocytes via co-culture with chondrocytes on hydrogel constructs for the repair of mandibular condylar cartilage defects
**49**	Platelet-rich plasma inhibits RANKL-induced osteoclast differentiation through activation of Wnt pathway during bone remodeling	Wang, DY. et al., 2018	15(3.75)	17(4.25)	31(7.75)	10.3892/ijmm. 2017.3258	*In vitro*	Evaluated the effect of platelet-rich plasma on the remodeling of the mandibular condyle

**50**	Temporomandibular Joint Regenerative Medicine	Van Bellinghen, X. et al., 2018	24 (6.00)	25 (6.25)	52 (13.00)	10.3390/ijms19020446	Literature review	Discussed the characteristics and etiology of TMD as well as proposals for therapies for the treatment of dysfunction aimed at regenerative treatments
**51**	Approaches to improve integration and regeneration of an ex vivo derived temporomandibular joint disc scaffold with variable matrix composition	Matuska, AM. et al., 2018	8(2.00)	8(2.00)	9(2.25)	10.1007/s10856-018-6164-z	*In vitro*	Evaluated decellularization, lipid removal, mechanical properties, ECM characteristics and initial interactions with resembed human mesenchymal stem cells in the TMJ disk fixation treatment
**52**	Inhibition of notch signaling pathway temporally postpones the cartilage degradation progress of temporomandibular joint arthritis in mice	Luo, XT. et al., 2018	14(3.50)	17(4.25)	17(4.25)	10.1016/j.jcms.2018.04.026	*In vivo*	Explored the role of the Notch signaling pathway in the initiation and progression of TMJ osteoarthritis
**53**	Effect of bone marrow mesenchymal stem cells on healing of temporomandibular joints in rats with induced rheumatoid arthritis	El Qashty, RMN. et al., 2018	10(2.50)	10(2.50)	13(3.25)	10.1111/eos.12533	*In vivo*	Evaluated the effect of bone marrow mesenchymal stem cells on TMJ repair with induced rheumatoid arthritis
**54**	The many faces of genetic contributions to temporomandibular joint disorder: An updated review	Scariot, R. et al., 2018	3(0.75)	3(0.75)	6(1.50)	10.1111/ocr.12239	Literature review	Discussed the genetic contributions to TMJ dysfunction

**55**	Profiling of Stem/Progenitor Cell Regulatory Genes of the Synovial Joint by Genome-Wide RNA-Seq Analysis	Zhou, Y. et al., 2018	4(1.00)	6(1.50)	8(2.00)	10.1155/2018/9327487	*In vitro*	Described the genome profiling and analyzing molecular signaling pathways relevant to stem/progenitor cell functions
**56**	Scaffold-free cartilage cell sheet combined with bone-phase BMSCs-scaffold regenerate osteochondral construct in mini-pig model	Wang, FY. et al., 2018	17(4.25)	18(4.50)	25(6.25)		*In vivo*	Analyzed the use of sheets of cartilage cells combined with a scaffold of mesenchymal stem cells from bone marrow to induce condylar regeneration
**57**	Scaffold-Based Temporomandibular Joint Tissue Regeneration in Experimental Animal Models: A Systematic Review	Helgeland, E. et al., 2018	18(4.50)	23(5.75)	33(8.25)	10.1089/ten.teb.2017.0429	Systematic review	Investigated whether the implantation of scaffolds of biomaterials loaded with cells and/or growth factors increases the regeneration of discal or osteochondral tissues of the TMJ, in comparison with scaffolds alone, without cells, or only growth factors
**58**	Physiology and Engineering of the Graded Interfaces of Musculoskeletal Junctions	Bonnevie, ED. et al., 2018	27 (6.75)	27 (6.75)	33 (8.25)	10.1146/annurev-bioeng-062117-121113	Literature review	Described the organization and structure of complex musculoskeletal interfaces, and new technologies for engineering these structures as the requirements for assessing the nature of these tissues in the context of recapitulating their function through tissue engineering

**59**	Applications of stem cells in orthodontics and dentofacial orthopedics: Current trends and future perspectives	Safari, S. et al., 2018	11(2.75)	15(3.75)	27(6.75)	10.4252/wjsc.v10.i6.66	Literature review	Investigated the current and future applications of stem cells in orthodontics and dentofacial orthopedics
**60**	Fibrocartilage Stem Cells Engraft and Self-Organize into Vascularized Bone	Nathan, J. et al., 2018	10(2.50)	10(2.50)	15(3.75)	10.1177/0022034517735094	*In vivo*	Explored the fibrocartilage stem cells and endothelial cell interactions during vascularized bone formation
**61**	Intra-articular biomaterials-assisted delivery to treat temporomandibular joint disorders	Dashnyam, K. et al., 2018	22 (5.50)	22 (5.50)	28 (7.00)	10.1177/2041731418776514	Literature review	Presented nano/microparticle systems used in intra-articular therapy to prevent cartilage degradation and protect the TMJ subchondral bone
**62**	Biomarker expression related to chondromatosis in the temporomandibular joint	Yoshitake, H. et al., 2019	1(0.33)	1(0.33)	4(1.33)	10.1080/08869634.2019.1622291	*In vitro*	Identified biomarkers for cell proliferation and chondrogenesis in the primary stage of synovial chondromatosis in TMJ and its results improve the understanding of the etiology and progression of the disease
**63**	An in situ hybridization study of MMP-2, -9, -13, -14, TIMP-1, and -2 mRNA in fetal mouse mandibular condylar cartilage as compared with limb bud cartilage	Takahashi, M. et al., 2019	3(1.00)	7(2.33)	Não encontrado	10.1016/j.gep.2019.02.003	*In situ*	Investigated mRNA expression of MMPs and TIMPs in developing mandibular condylar cartilage

**64**	Inhibition of Ihh Reverses Temporomandibular Joint Osteoarthritis via a PTH1R Signaling Dependent Mechanism	Yang, HX. et al., 2019	24 (8.00)	25 (8.33)	29 (9.67)	10.3390/ijms20153797	*In vitro/in vivo*	Analyzed the relationship between the Ihh protein and the signaling of the parathyroid hormone receptor 1 in the modulation of the terminal differentiation of chondrocytes in the osteoarthritic cartilage of the TMJ
**65**	Effect of human adipose-derived regenerative cells on temporomandibular joint healing in immunodeficient rabbits	Putnova, B. et al., 2019	0	1(0.33)	1(0.33)	10.2754/avb201988010049	*In vivo*	Evaluated the effect of the application of adipose tissue stem cells in the healing of TMJ tissues, for possible use in the treatment of TMD
**66**	Role of Link N in Modulating Inflammatory Conditions	Yang, MC. et al., 2019	1(0.33)	1(0.33)	1(0.33)	10.11607/ofph.1952	*In vitro/ in vivo*	Clarified the role of Link N in the regulation of inflammatory molecules of human mesenchymal stem cells under interleukin (IL)-1β stimulation and showed that it is efficient in modulating inflammation
**67**	Regenerative Effect of Platelet Concentrates in Oral and Craniofacial Regeneration	Al-Hamed, FS. et al., 2019	17(5.67)	20(6.67)	35(11.67)	10.3389/fcvm.2019.00126	*Revisão*	Review the clinical applications of platelet concentrates in oral and craniofacial tissue regeneration and the role of their molecular components in tissue healing

**68**	Combination of polyetherketoneketone scaffold and human mesenchymal stem cells from temporomandibular joint synovial fluid enhances bone regeneration	Lin, Y. et al., 2019	20 (6.67)	24 (8.00)	33 (11.00)	10.1038/s41598-018-36778-2	*In vitro*	Analyzed the adhesion of mesenchymal stem cells from synovial fluid to polyetherketonacetone scaffolds and their osteogenic potential
**69**	Therapeutic effect of mesenchymal stem cells derived from human umbilical cord in rabbit temporomandibular joint model of osteoarthritis	Kim, H. et al., 2019	17(5.67)	18(6.00)	26(8.67)	10.1038/s41598-019-50435-2	*In vivo*	Investigated the cartilage regenerating and anti-inflammatory effects of mesenchymal stem cells from human umbilical cord matrix at different concentrations for the treatment of TMJ osteoarthritis
**70**	Protective effects of extracorporeal shockwave on rat chondrocytes and temporomandibular joint osteoarthritis; preclinical evaluation with in vivo Tc-99m-HDP SPECT and ex vivo micro-CT	Kim, YH. et al., 2019	18(6.00)	Não encontrado	26(8.67)	10.1016/j.joca.2019.07.008	*In vivo*	Investigated the effects of extracorporeal shock wave therapy on TMJ osteoarthritis using chondrocytes in a model of TMJ osteoarthritis
**71**	Voluntary biting behavior as a functional measure of orofacial pain in mice	Guo, W. et al., 2019	6(2.00)	6(2.00)	9(3.00)	10.1016/j.physbeh.2019.02.024	*In vivo*	Evaluated the effect of bone marrow stromal cell infusion for relief of orofacial pain and involuntary bite induced by masseter muscle tendon ligation injury

**72**	Temporomandibular Disorders Clinical and Anatomical Outcomes After Fat-Derived Stem Cells Injection	Carboni, A. et al., 2019	6(2.00)	6(2.00)	15(5.00)	10.1097/SCS.0000000000004884	Clinical trial study	Analyzed the effect of fat-derived stem cell injection into the joint as a new treatment option
**73**	Knockdown of long non-coding RNA AK094629 attenuates the interleukin-1 beta induced expression of interleukin-6 in synovium-derived mesenchymal stem cells from the temporomandibular joint	Jia, JX. et al., 2020	4(2.00)	Não encontrado	5(2.50)	10.3892/mmr.2020.11193	*In vitro*	Investigated the effect of AK094629 on IL-1β-induced IL-6 expression in TMJ synovial mesenchymal stem cells
**74**	LOXL2 promotes aggrecan and gender-specific anabolic differences to TMJ cartilage	Tashkandi, MM. et al., 2020	2(1.00)	2(1.00)	3(1.50)	10.1038/s41598-020-77178-9	*In vivo*	Evaluated in an animal osteoarthritis model whether LOXL2 attenuates pro-inflammatory signaling and induces chondroprogenitor and regenerative response
**75**	Electrospun Polymers in Cartilage Engineering-State of Play	Yilmaz, EM. et al., 2020	15(7.50)	18(9.00)	24(12.00)	10.3389/fbioe.2020.00077	Literature review	Described the structure of cartilage, the main modulators of chondrogenesis and advances in the use of scaffolds
**76**	Effects of dynamic radial tensile stress on fibrocartilage differentiation of bone marrow mesenchymal stem cells	Su, XL. et al., 2020	5(2.50)	6(3.00)	7(3.50)	10.1186/s12938-020-0751-1	*In vitro*	Evaluated the power of differentiation of mesenchymal stem cells from bone marrow, presenting a new method for differentiating these cells into fibrochondrocytes

**77**	Rapamycin-Induced Autophagy Promotes the Chondrogenic Differentiation of Synovium-Derived Mesenchymal Stem Cells in the Temporomandibular Joint in Response to IL-1 beta	Liu, WJ. et al., 2020	5(2.50)	5(2.50)	7(3.50)	10.1155/2020/4035306	*In vitro*	Investigated the capacity and mechanisms of synovial mesenchymal stem cells in the inflammatory microenvironment of TMJ
**78**	Photobiomodulation and Stem Cell Therapy for Temporomandibular Joint Disc Disorders	Karic, V. et al., 2020	3(1.50)	3(1.50)	5(2.50)	10.1089/photob.2019.4790	Literature review	Reported updated informations about photobiomodulation and stem cells as an alternative treatment for TMJ disc degeneration
**79**	Conditional deletion of Adrb2 in mesenchymal stem cells attenuates osteoarthritis-like defects in temporomandibular joint	Sun, JL. et al., 2020	10(5.00)	10(5.00)	14(7.00)	10.1016/j.bone.2020.115229	*In vivo*	Evaluation of mesenchymal stem cells derived from subchondral bone in the presence of Adrb2 expression attenuates alterations caused by TMJ osteoarthritis
**80**	Suberoylanilide Hydroxamic Acid Attenuates Interleukin-1 beta-Induced Interleukin-6 Upregulation by Inhibiting the Microtubule Affinity-Regulating Kinase 4/Nuclear Factor-kappa B Pathway in Synovium-Derived Mesenchymal Stem Cells from the Temporomandibular Joint	Sun, JD. et al., 2020	8(4.00)	Não encontrado	10(5.00)	10.1007/s10753-020-01204-1	*In vivo*	Evaluated the effect of suberoylanilide hydroxamic acid on IL-1β-induced synovial mesenchymal stem cell IL-6 attenuation, also examining the role of microtubules4 (MARK4) in the process

**81**	Factors secreted from dental pulp stem cells show multifaceted benefits for treating experimental temporomandibular joint osteoarthritis	Ogasawara, N. et al., 2020	19 (9.50)	19 (9.50)	29 (14.50)	10.1016/j.joca. 2020.03.010	*In vitro*	Investigated effective therapies using stem cells extracted from deciduous teeth with the aim of reversing or suppressing the progression of TMJ osteoarthritis
**82**	Anti-Inflammatory Effect of Adipose-Derived Stromal Vascular Fraction on Osteoarthritic Temporomandibular Joint Synoviocytes	Kim, H. et al., 2020	7(3.50)	7(3.50)	11(5.50)	10.1007/s1377 0-020-00268-2	*In vitro*	Demonstrated the use of vascular stromal fraction containing stem cells derived from adipose tissue for the treatment of TMJ osteoarthritis
**83**	DPSCs Attenuate Experimental Progressive TMJ Arthritis by Inhibiting the STAT1 Pathway	Cui, SJ. et al., 2020	11(5.50)	9(4.50)	18(9.00)	10.1177/0022 034520901710	*In vivo*	Investigated the therapeutic effects of human dental pulp stem cells in TMJ arthritis
**84**	MicroRNA-29b Promotes Subchondral Bone Loss in TMJ Osteoarthritis	Sun, JL. et al., 2020	9(4.50)	8(4.00)	10(5.00)	10.1177/0022 034520937617	*In vivo*	Evaluated miR29 expression in mesenchymal stem cells from bone marrow and its effect on bone remodeling
**85**	Notch Regulates Fibrocartilage Stem Cell Fate and Is Upregulated in Inflammatory TMJ Arthritis	Ruscitto, A. et al., 2020	6(3.00)	7(3.50)	8(4.00)	10.1177/0022 034520924656	*In vivo*	Investigated the role of the Notch pathway in regulating TMJ development and the fate of fibrocartilage stem cells

**86**	Angiostatin-functionalized collagen scaffolds suppress angiogenesis but do not induce chondrogenesis by mesenchymal stromal cells in vivo	Helgeland, E. et al., 2020	1(0.50)	2(1.00)	11(0.50)	10.2334/josnusd.19-0327	*In vivo*	Used stromal mesenchymal stem cells loaded on scaffolds to investigate the effect of angiostatin in inhibiting angiogenesis and promoting chondrogenesis
**87**	Tissue engineered autologous cartilage-bone grafts for temporomandibular joint regeneration	Chen, D. et al., 2020	23 (11.50)	25 (12.50)	31 (15.50)	10.1126/scitranslmed.abb6683	*In vivo*	Demonstrated the feasibility of TMJ regeneration by performing specific tissue transplants
**88**	Differences in the biological properties of mesenchymal stromal cells from traumatic temporomandibular joint fibrous and bony ankylosis: a comparative study	Zhang, PP. et al., 2021	1(1.00)	1(1.00)	1(1.00)	10.1080/19768354.2021.1978543	*In vitro*	Analyzed the functional characteristics of mesenchymal stromal cells from a model of traumatic fibrous and osseous ankylosis of the TMJ
**89**	Enhanced Circadian Clock in MSCs-Based Cytotherapy Ameliorates Age-Related Temporomandibular Joint Condyle Degeneration	Cha, S. et al., 2021	1(1.00)	1(1.00)	1(1.00)	10.3390/ijms221910632	*In vivo*	Investigated the age-related morphology and changes in molecular and circadian rhythms in TMJ condyles and illustrate their relationship with TMJ degeneration
**90**	Temporomandibular Joint Osteoarthritis: Regenerative Treatment by a Stem Cell Containing Advanced Therapy Medicinal Product (ATMP)-An In Vivo Animal Trial	Kohnke, R. et al., 2021	5(5.00)	6(6.00)	7(7.00)	10.3390/ijms22010443	*In vivo*	Compared the chondrogenic capacity of therapy with intra-articular stromal stem cells and serum and hyaluronic acid

**91**	Dual-crosslinked 3D printed gelatin scaffolds with potential for temporomandibular joint cartilage regeneration	Helgeland, E. et al., 2021	6(6.00)	7(7.00)	7(7.00)	10.1088/1748-605X/abe6d9	*In vitro*	Evaluated non-toxic crosslinking methods to improve the physical properties of gelatin scaffolds for cartilage regeneration
**92**	Human Umbilical Cord Mesenchymal Stem Cells Over Platelet Rich Fibrin Scaffold for Mandibular Cartilage Defects Regenerative Medicine	Sumarta, NPM. et al., 2021	0	1(1.00)	1(1.00)	10.1590/pboci.2021.020	*In vivo*	Assessed whether there was regeneration of the mandibular fibrocartilage defect after implantation of mesenchymal stem cells on platelet-rich fibrin, which proved to be efficient when there is expression of FGF 18, Sox9, Ki67
**93**	BMSC-Derived Small Extracellular Vesicles Induce Cartilage Reconstruction of Temporomandibular Joint Osteoarthritis via Autotaxin-YAP Signaling Axis	Wang, YN. et al., 2021	4(4.00)	5(5.00)	5(5.00)	10.3389/fcell.2021.656153	*In vitro*	Analyzed the effect of cartilage reconstruction with bone marrow mesenchymal stem cells on TMJ and the mechanism underlying this effect
**94**	Elder Mice Exhibit More Severe Degeneration and Milder Regeneration in Temporomandibular Joints Subjected to Bilateral Anterior Crossbite	Zhang, YJ. et al., 2021	1(1.00)	1(1.00)	1(1.00)	10.3389/fphys.2021.750468	*In vivo*	Based on an animal crossbite model, the effect of aging on degenerative remodeling in the TMJ condyles in response to biomechanical stimulation was evaluated. The decrease in the amount of cells was considered one of the effects of aging

**95**	Fibrocartilage Stem Cells in the Temporomandibular Joint: Insights From Animal and Human Studies	Fan, Y. et al., 2021	4(4.00)	4(4.00)	4(4.00)	10.3389/fcell. 2021.665995	Literature review	Presented the characteristics and functions of TMJ fibrocartilage stem cells and their therapeutic applications
**96**	An Update on Mesenchymal Stem Cell-Centered Therapies in Temporomandibular Joint Osteoarthritis	Zhao, YF. Et al., 2021	4(4.00)	5(5.00)	6(6.00)	10.1155/2021/ 6619527	Literature review	Described the therapeutic roles of mesenchymal stem cells, exosomes and stromal vascular fraction in osteoarthritis as well as the pathological mechanism of the therapies
**97**	Long non-coding RNA XIST regulates chondrogenic differentiation of synovium-derived mesenchymal stem cells from temporomandibular joint via miR-27b-3p/ADAMTS-5 axis	Zhu, Y. et al., 2021	6(6.00)	6(6.00)	8(8.00)	10.1016/j.cyto .2020.155352	*In vitro*	Investigated the role of XIST in the chondrogenic differentiation of human synovial mesenchymal stem cells from TMJ
**98**	A bilayered scaffold with segregated hydrophilicity-hydrophobicity enables reconstruction of goat hierarchical temporomandibular joint condyle cartilage	Yu, X. et al., 2021	8(8.00)	8(8.00)	8(8.00)	10.1016/j.actbi o.2020.11.031	*In vivo*	Evaluated the regeneration of condylar cartilage through the use of a two-layer scaffold with hydrophilicity-hydrophobicity and its effects on stem cell differentiation

**99**	Cell-mediated injectable blend hydrogel-BCP ceramic scaffold for in situ condylar osteochondral repair	Wang, HZ. et al., 2021	8(8.00)	10(10.00)	12(12.00)	10.1016/j.actbio.2020.12.056	*In situ*	Evaluated the cartilaginous regeneration through the injection of hydrogel HA-SH/Col I encapsulated with mesenchymal stem cells and chondrocytes
**100**	Conditioned medium from mesenchymal stem cells improves condylar resorption induced by mandibular distraction osteogenesis in a rat model	Katagiri, W. et al., 2021	1(1.00)	1(1.00)	1(1.00)	10.1016/j.heliyon.2021.e06530	*In vivo*	The effects of bone marrow mesenchymal stem cells on condylar resorption were investigated, showing high osteogenic, chondrogenic and angiogenic activity
**101**	The efficacy of mesenchymal stem cells in regenerating structures associated with the temporomandibular joint: A systematic review	Pagotto, LEC. et al., 2021	2(2.00)	2(2.00)	2(2.00)	10.1016/j.archoralbio.2021.105104	Systematic review	Evaluated mesenchymal stem cells as a promising strategy in the regenerative treatment of TMJ resorption
**102**	Overexpression of HIF-1alpha in Bone Marrow Mesenchymal Stem Cells Promote the Repair of Mandibular Condylar Osteochondral Defect in a Rabbit Model	Cheng, MS. et al., 2021	2(2.00)	4(4.00)	5(5.00)	10.1016/j.joms.2020.10.013	*In vivo*	Evaluated the effect of HIF-1 alpha overexpression in bone marrow mesenchymal stem cells aggregated to collagen scaffolds for the repair of TMJ condylar osteochondral defects

**103**	Mesenchymal Stromal Cell Transplantation Induces Regeneration of Large and Full-Thickness Cartilage Defect of the Temporomandibular Joint	Gomez, M. et al., 2021	8(8.00)	8(8.00)	10(10.00)	10.1177/1947603520926711	*In vivo*	Evaluated the ability of mesenchymal stromal cells to regenerate TMJ cartilage
**104**	The Role of TNF-alpha in the Pathogenesis of Temporomandibular Disorders	Wang, YR. et al., 2021	2(2.00)	2(2.00)	2(2.00)	10.1248/bpb.b21-00154	Literature review	Explored the general mechanisms of TNF-α, and the pathways mediated by it, as well as its roles in the treatment of TMD
**105**	Molecular signaling in temporomandibular joint osteoarthritis	Lu, K. et al., 2022	6	6	6	10.1016/j.jot.2021.07.001	Literature review	Discussed the signaling pathways involved in the development and progression of TMJ osteoarthritis and therapeutic strategies using stem cells
**106**	Autologous Stem Cells Transplants in the Treatment of Temporomandibular Joints Disorders: A Systematic Review and Meta-Analysis of Clinical Trials	Checinski, M. et al., 2022	1	1	1	10.3390/cells11172709	Systematic review	Analyzed the treatment of TMJ joint pain and restriction of maximum opening through the administration of mesenchymal stem cells from the evaluation of clinical studies
**107**	Mesenchymal Stem Cells Based Treatment in Dental Medicine: A Narrative Review	Smojver, I. et al., 2022	2	2	3	10.3390/ijms23031662	Literature review	Discussed about the use of stem cells in the field of regenerative dentistry

**108**	Effects of interleukin 1 beta on long noncoding RNA and mRNA expression profiles of human synovial fluid derived mesenchymal stem cells	Sun, YP. et al., 2022	0	Não encontrado	0	10.1038/s41598-022-12190-9	*In vitro*	Evaluated the profiles of lncRNA and mRNA expression of IL-1β-stimulated synovial fluid mesenchymal stem cells to identify potential therapeutic targets
**109**	Synovial macrophages in cartilage destruction and regeneration-lessons learnt from osteoarthritis and synovial chondromatosis	Li, YJ. et al., 2022	1	2	3	10.1088/1748-605X/ac3d74	Literature review	Addressed the role of macrophage polarization in the process of cartilage destruction and regeneration and the influence of the macrophage present in an inflammatory environment on cartilage fate
**110**	Long-term effect of bilateral anterior elevation of occlusion on the temporomandibular joints	Zhang, YJ. et al., 2022	4	4	6	10.1111/odi.13914	*In vitro*	Evaluated the effect of bilateral anterior elevation on TMJs
**111**	Regeneration of temporomandibular joint using in vitro human stem cells: A review	Gong, S. et al., 2022	2	4	4	10.1002/term.3302	Literature review	Reviewed the potential of several human stem cells for TMJ regeneration and suggests that further study be carried out to ensure the effectiveness of these treatments

**112**	Superwettable and injectable GelMA-MSC microspheres promote cartilage repair in temporomandibular joints	Yang, Y. et al., 2022	0	0	0	10.3389/fbioe.2022.1026911	*In vitro*	Analyzed the ability of mesenchymal stem cells to infiltrate the surface of GelMA microspheres loaded with TGFβ, leading to active infiltration of the biomaterial
**113**	940 nm diode laser induced differentiation of human adipose derived stem cells to temporomandibular joint disc cells	Karic, V. et al., 2022	0	0	0	10.1186/s12896-022-00754-6	*In vitro*	Examined the ability of human adipose tissue stem cells to differentiate into fibroblasts and chondrocytes using a 940 nm diode laser for possible TMD therapy
**114**	GDF11 inhibits abnormal adipogenesis of condylar chondrocytes in temporomandibular joint osteoarthritis	Wang, H. et al., 2022	1	1	1	10.1302/2046-3758.117.BJR-2022-0019.R1	*In vivo*	Demonstrated the involvement of growth and differentiation factor 11 in the differentiation of stem cells, however the study investigates whether this factor is involved in the abnormal adipogenesis of TMJ chondrocytes
**115**	Histone deacetylase inhibitors attenuated interleukin-1 beta-induced chondrogenesis inhibition in synovium-derived mesenchymal stem cells of the temporomandibular joint	Liao, W. et al., 2022	1	Não encontrado	1	10.1302/2046.3758.111.BJR-2021-0059.R1	*In vitro/ in vivo*	Explained the mechanism of histone deacetylase inhibitors and confirmed that they improved chondrogenesis inhibited by IL-1β

**116**	PTHrP promotes subchondral bone formation in TMJ-OA	Zhang, J. et al., 2022	0	0	0	10.1038/s41368-022-00189-x	*In vivo*	Based on an animal model of osteoarthritis, the study evaluated the role of parathyroid hormone in cartilage regeneration
**117**	Temporomandibular Joint Fibrocartilage Contains CD105 Positive Mouse Mesenchymal Stem/Progenitor Cells with Increased Chondrogenic Potential	Thamm, JR. et al., 2022	0	1	1	10.1007/s12663-022-01721-6	*In vivo*	Identified and characterized a subset of progenitor mesenchymal stem cells in TMJ cartilage that when activated can aid in fibrocartilage repair
**118**	Intra-articular injection of a novel Wnt pathway inhibitor, SM04690, upregulates Wnt16 expression and reduces disease progression in temporomandibular joint osteoarthritis	Hua, BQ. et al., 2022	3	3	5	10.1016/j.bone.2022.116372	*In vivo*	Based on a model of TMJ osteoarthritis induced by partial disk discectomy, SM04690 was evaluated for its effects on cartilage protection and regeneration
**119**	MicroRNA-26b regulates BMSC osteogenic differentiation of TMJ subchondral bone through ll-catenin in osteoarthritis	Yang, JL. et al., 2022	0	Não encontrado	0	10.1016/j.bone.2022.116448	*In vitro*	In an osteoarthritis model, the study showed that the level of miR-26b can modulate subchondral bone loss and influence osteogenic differentiation

**120**	Mesenchymal stem cells-derived microvesicles versus platelet-rich plasma in the treatment of monoiodoacetate-induced temporomandibular joint osteoarthritis in Albino rats	AbuBakr, N. et al., 2022	0	0	0	10.1016/j.heliyon.2022.e10857	*In vivo*	This study compared the result of a single intra-articular injection of microvesicles derived from bone marrow mesenchymal stem cells versus a single injection of platelet-rich pasma in monoiodoacetate and evaluated its curative potential in TMJ joint osteoarthritis
**121**	Stem Cells in Temporomandibular Joint Engineering: State of Art and Future Persectives	Minervini, G. et al., 2022	1	1	3	10.1097/SCS.0000000000008771	Literature review	Described the possible sources of mesenchymal stem cells and new approaches for TMJ cartilage regeneration
**122**	Inhibiting Hh Signaling in Gli1(+) Osteogenic Progenitors Alleviates TMJOA	Lei, J. et al., 2022	2	2	3	10.1177/00220345211059079	*In vivo*	Studied the role of Gli1+ cells in an animal model of osteoarthritis, showing that they contribute to the formation of subchondral bone
**123**	Mesenchymal Stem Cell Exosomes Promote Functional Osteochondral Repair in a Clinically Relevant Porcine Model	Zhang, SP. et al., 2022	6	6	8	10.1177/03635465211068129	*In vivo*	Evaluated the effectiveness of human mesenchymal stem cell exosomes in osteochondral repair

**124**	Regulating Fibrocartilage Stem Cells via TNF-alpha/Nf-kappa B in TMJ Osteoarthritis	Bi, R. et al., 2022	4	6	7	10.1177/0022034521103724 8	*In vitro*	Investigated the abilities of fibrocartilage stem cells to regulate tumor necrosis factor α signaling for cartilage repair in TMJ osteoarthritis
**125**	Inflammation-Stimulated MSC-Derived Small Extracellular Vesicle miR-27b-3p Regulates Macrophages by Targeting CSF-1 to Promote Temporomandibular Joint Condylar Regeneration	Liu, YF. et al., 2022	3	2	3	10.1002/smll.202107354	*In vitro/ in vivo*	Used acellular osteochondral scaffolds with controlled release of IAE and evaluated the efficiency of phenotypic changes in macrophages to promote repair of condylar osteochondral defect and the associated mechanism

Supplementary file 2: The 125 papers on the directions of Temporomandibular Disorders and Stem Cells
